# Twisted Intramolecular Charge Transfer State of a “Push-Pull” Emitter

**DOI:** 10.3390/ijms21217999

**Published:** 2020-10-27

**Authors:** Sebok Lee, Myungsam Jen, Yoonsoo Pang

**Affiliations:** Department of Chemistry, Gwangju Institute of Science and Technology, 123 Cheomdangwagi-ro, Buk-gu, Gwangju 61005, Korea; leesebok@gist.ac.kr (S.L.); watqdt@gist.ac.kr (M.J.)

**Keywords:** intramolecular charge transfer, excited-state dynamics, femtosecond stimulated raman spectroscopy, push-pull emitter, twisted intramolecular charge transfer

## Abstract

The excited state Raman spectra of 4-dicyanomethylene-2-methyl-6-(*p*-dimethylaminostyryl)-4*H*-pyran (DCM) in the locally-excited (LE) and the intramolecular charge transfer (ICT) states have been separately measured by time-resolved stimulated Raman spectroscopy. In a polar dimethylsulfoxide solution, the ultrafast ICT of DCM with a time constant of 1.0 ps was observed in addition to the vibrational relaxation in the ICT state of 4–7 ps. On the other hand, the energy of the ICT state of DCM becomes higher than that of the LE state in a less polar chloroform solution, where the initially-photoexcited ICT state with the LE state shows the ultrafast internal conversion to the LE state with a time constant of 300 fs. The excited-state Raman spectra of the LE and ICT state of DCM showed several major vibrational modes of DCM in the LE and ICT conformer states coexisting in the excited state. Comparing to the time-dependent density functional theory simulations and the experimental results of similar push-pull type molecules, a twisted geometry of the dimethylamino group is suggested for the structure of DCM in the S_1_/ICT state.

## 1. Introduction

Intramolecular charge transfer (ICT) process has been of great interest in chemistry and related disciplines for many decades [1,2,3,4,5,6,7,8,9]. The ICT is considered as the first step of the charge-separation processes in natural photosynthesis, where solar energy is converted into chemical energy. The dyes with strong ICT are often widely used in fluorescence applications due to many photophysical advantages. 4-Dicyanomethylene-2-methyl-6-(*p*-dimethylaminostyryl)-4*H*-pyran (DCM) is one of the “push-pull” emitters, where a strong ICT occurs between the electron donor (dimethylamino; DMA) and the electron acceptor (dicyanomethylene) connected by a π-bridge [10,11,12,13,14,15,16]. Due to the distinct photophysical aspects, DCM has been used in numerous applications including laser dyes [15,16], OLED emitters [17,18], molecular photo-switches [19,20,21,22].

Despite the wide applications of the ICT process of the “push-pull” emitters, the details of the ICT process including the structural changes were not clearly understood yet. The “twisted” ICT (TICT) state with a perpendicular conformation of the donor group to the molecular plane [23,24,25,26,27] and the “planar” ICT (PICT) [11,13] with the parallel conformation between the donor and acceptor groups have been proposed by numerous theoretical approaches [25,27,28,29,30]. Millie and co-workers suggested a TICT model with the perpendicularly twisted DMA group for DCM by conformation spectra-intermediate neglect of differential overlap (CS INDO) multi references configuration interaction (MRCI) simulations [28]. Similarly, a TICT state along the dimethylaminophenyl (DMAP) rotation was obtained by the complete active space self-consistent-field (CASSCF) calculations [27]. On the other hand, the planar structures of DCM in both the ground state and the excited state were proposed by time-dependent density functional theory (TDDFT) simulations with the CAM-B3LYP functional [30]. Recently, another TDDFT study at the mPW1PBE/6-31G(d) level of theory suggested the TICT state with a twisted DMAP group for the emitting S_1_ state of DCM in ethanol [29]. The theoretical investigation for the molecular geometry in the excited state strongly depends on the choice of various first-principle approaches. Even with the TDDFT simulations considered as the most efficient computation method for the excited states, the choice of approximate exchange-correlational functional is very important for the accuracy of the simulation results [31].

**Scheme 1:**
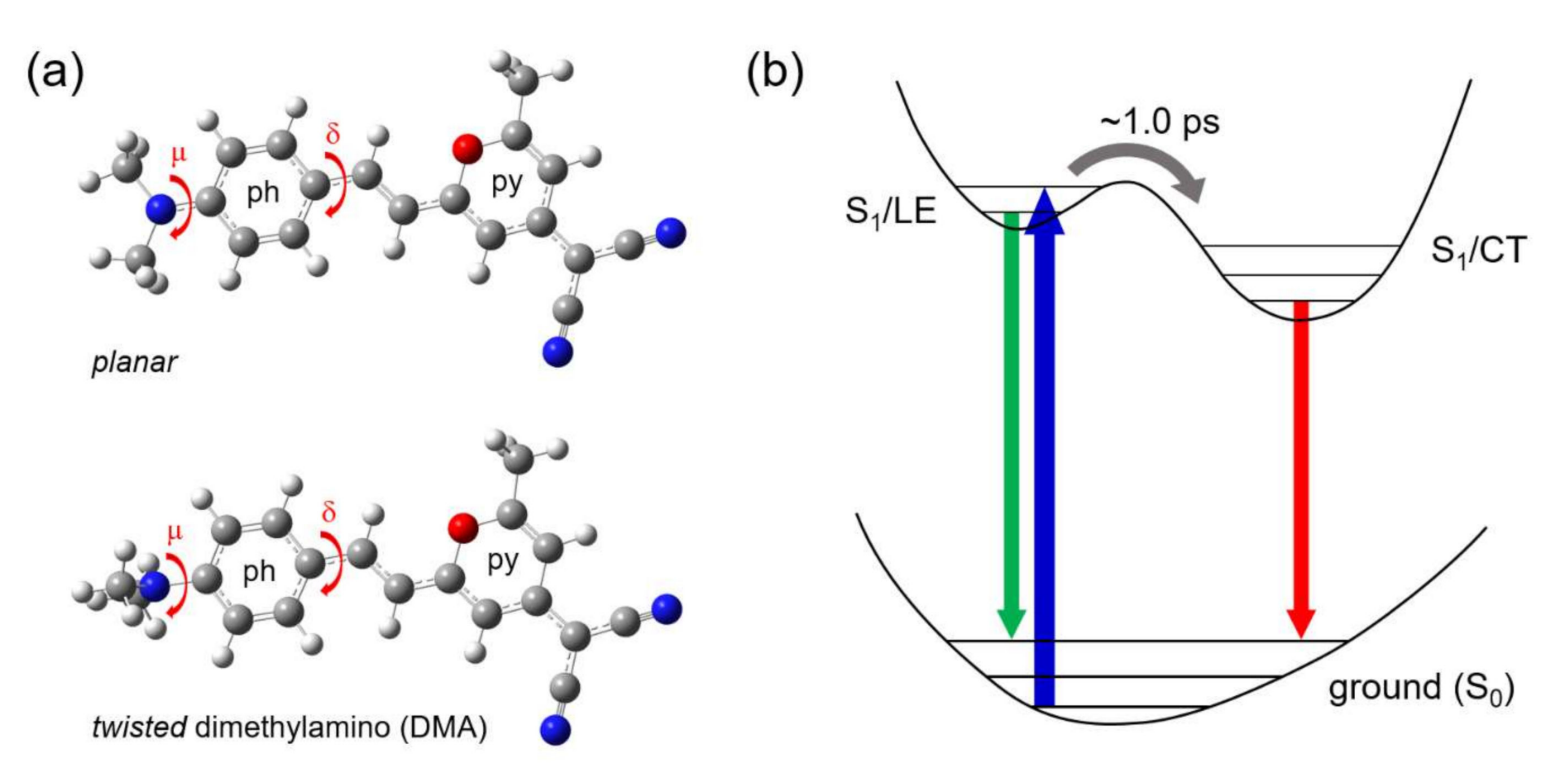
(**a**) Molecular structures of 4-dicyanomethylene-2-methyl-6-(p-dimethylaminostyryl)-4H-pyran (DCM) with plausible structural changes upon the intramolecular charge transfer, (**b**) summary of the photophysics of DCM in polar solvents. Planar and twisted dimethylamino structures were optimized in the time-dependent density functional theory (TDDFT) simulations at the B3LYP/6-311G(d,p) level.

No straightforward experimental evidence for the structure of the ICT state of DCM or similar push-pull dyes have been reported by now [10,11,12,13,14,25,32,33,34,35,36,37,38]. Numerous investigations on the excited-state dynamics of DCM by using the time-resolved transient absorption and fluorescence spectroscopy have been made [11,12,13,14,25,34]. However, the dynamics of the excited state absorption or emission may not provide enough information on the structural changes of molecules during the ICT process. Fleming and co-workers used femtosecond mid-infrared absorption spectroscopy and two-dimensional electronic-vibrational spectroscopy for the study of the ICT process of DCM in dimethylsulfoxide (DMSO) and acetonitrile [32,33]. They found a strong infrared band at 1475 (or 1480) cm^−1^ exhibited a strong blue-shift of 10–12 cm^−1^ with the time constants of 270 fs and 2.9 ps, which is considered as the vibrational relaxation before and after the LE → ICT barrier crossing, respectively [32]. However, these experimental results using the infrared absorption probe only provided a limited number of vibrational modes, which seems to be incomplete for the explanation of the structural changes of molecules during the ICT process in the excited state. The proposed structural changes of DCM upon the ICT and the overall photophysics of DCM observed in most of the experimental and theoretical studies are summarized in Scheme 1.

Since Mathies and co-workers first introduced the femtosecond stimulated Raman spectroscopy (FSRS) to the ultrafast dynamics and transient Raman measurements of all-trans-β-carotene in the S_2_ and S_1_ excited states [39,40], FSRS with both high spectral (<10 cm^−1^) and temporal (<50 fs) resolutions has been widely applied to many excited state processes including the intra- and inter-molecular proton transfers [41,42,43,44], charge transfers [45,46,47,48], electron transfers [49,50,51,52]. A broadband Raman probe pulses combined with a narrowband Raman pump were used for FSRS, and the transient Raman bands of the ground and excited electronic states can be obtained at the same time in a wide frequency range covering most of the fingerprint region (800–2200 cm^−1^) [43,53]. Recently, McCamant and co-workers reported the FSRS measurements on 4-(dimethylamino)benzonitrile (DMABN), where the ultrafast ICT dynamics with the twist of the DMA group was clearly shown [46]. 

In this paper, we obtained the distinct Raman spectra of DCM in the LE and ICT excited states separately, by using an FSRS technique. The excited-state dynamics of the LE and ICT Raman bands of DCM in a polar DMSO and a less polar CHCl_3_ solvent has provided a clear explanation of the excited-state structure and the ICT dynamics. With these experimental vibrational results, the accurate molecular structures of DCM in the LE and ICT states will be sought by the DFT/TDDFT simulations.

## 2. Results

### 2.1. Steady-State Absorption and Emission Spectra of DCM 

**Figure 1:**
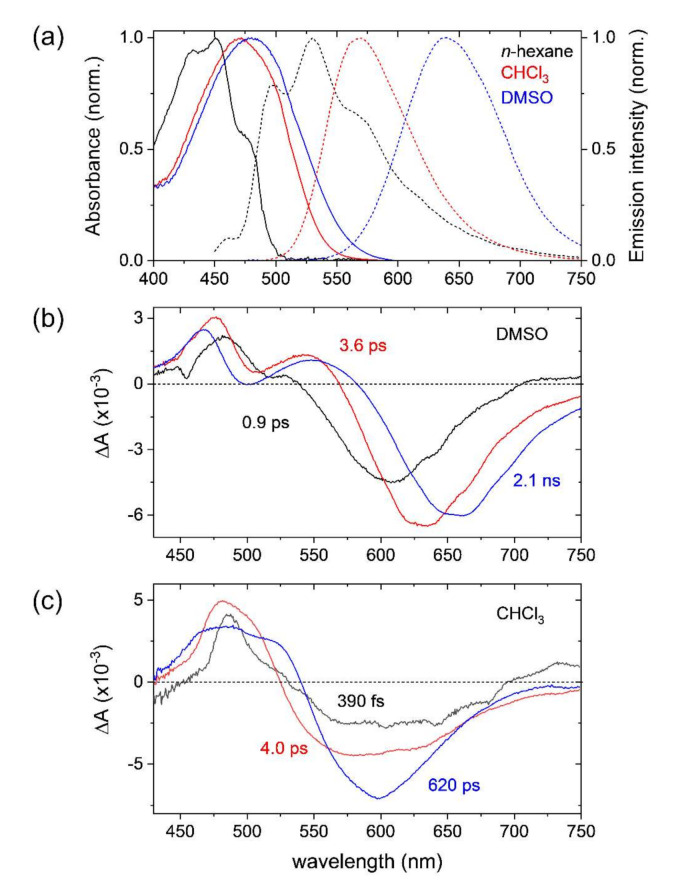
(**a**) Absorption (solid lines) and emission (dotted lines) spectra of DCM in *n*-hexane, CHCl_3_, and dimethylsulfoxide (DMSO) solutions. The excitations at 405 (*n*-hexane) and 485 nm (CHCl_3_ and DMSO) were used for the emission measurements; the evolution associated difference spectra (EADS) of DCM in (**b**) DMSO and (**c**) CHCl_3_ solution for the transient absorption results obtained with 403 nm excitation.

The steady-state absorption and emission spectra of DCM dissolved in *n*-hexane, chloroform (CHCl_3_), and dimethylsulfoxide (DMSO) are shown in Figure 1a. The absorption and emission maxima of DCM in nonpolar *n*-hexane appear at 451 and 530 nm, respectively, with well-resolved vibronic bands. The absorption band of DCM shows minor red-shifts in weakly polar CHCl_3_ (471 nm) and polar DMSO (480 nm) solutions from the nonpolar band position. The emission band of DCM appears at 639 nm in DMSO with a strong Stokes shift of 5230 cm^−1^. The increase of Stokes shifts from those in nonpolar *n*-hexane (3300 cm^−1^) and less polar CHCl_3_ (3580 cm^−1^) solutions confirms the ICT formation in the excited state in polar solvents, which shows a strong solvent polarity dependence known as the Lippert–Mataga model [12,22,32,33,54].

### 2.2. Transient Absorption Spectra and Kinetics of DCM 

Transient absorption measurements with 403 nm excitation were performed to study the ICT dynamics in the excited state of DCM in DMSO and CHCl_3_ solutions. Three kinetic components of 0.9, 3.6 ps, and 2.1 ns were obtained in the global analysis of the transient absorption results of DCM in the DMSO solution by applying a sequential decay model [55]. The evolution associated difference spectra (EADS) of three kinetic components obtained from DMSO solution are shown in Figure 1b: the fastest 0.9 ps component represents the ICT process of the LE state, and the 3.6 ps the vibrational relaxation of the ICT state, and the 2.1 ns the population decay of the ICT state of DCM. The assignment of three EADS is well supported by the difference in the excited state absorption and stimulated emission spectrum of DCM [22]. The ICT dynamics of DCM in DMSO with a 0.9 ps time constant appeared consistent with previous results. It is also interesting to note that the ICT dynamics are strongly dependent on the solvent polarity [12,13,33]. A much faster ~150 fs time constant has been reported for the ICT in a polar protic methanol solution by transient absorption and fluorescence upconversion measurements while the ultrafast ICT dynamics were not observed in less polar solvents.

Similarly, three kinetic components of 390 fs, 4.0 ps, and 620 ps were obtained for the DCM in CHCl_3_ solution as the corresponding EADS are shown in Figure 1c. The 4.0 ps and 620 ps components can be assigned with ease to the vibrational relaxation and the population decay of the LE state since the formation of ICT state in CHCl_3_ solution was not observed in previous studies [13,54]. However, it is not clear which state represents the fastest 390 fs component, which will be identified by time-resolved Raman measurements. Moreover, the absorption and emission features of these three components appear very similar to each other with minor differences in the bandwidth or shape, which in general represents the limitation of transient absorption measurements for the investigation of the photoinduced excited-state processes.

### 2.3. Time-Resolved Stimulated Raman Spectra of DCM 

**Figure 2:**
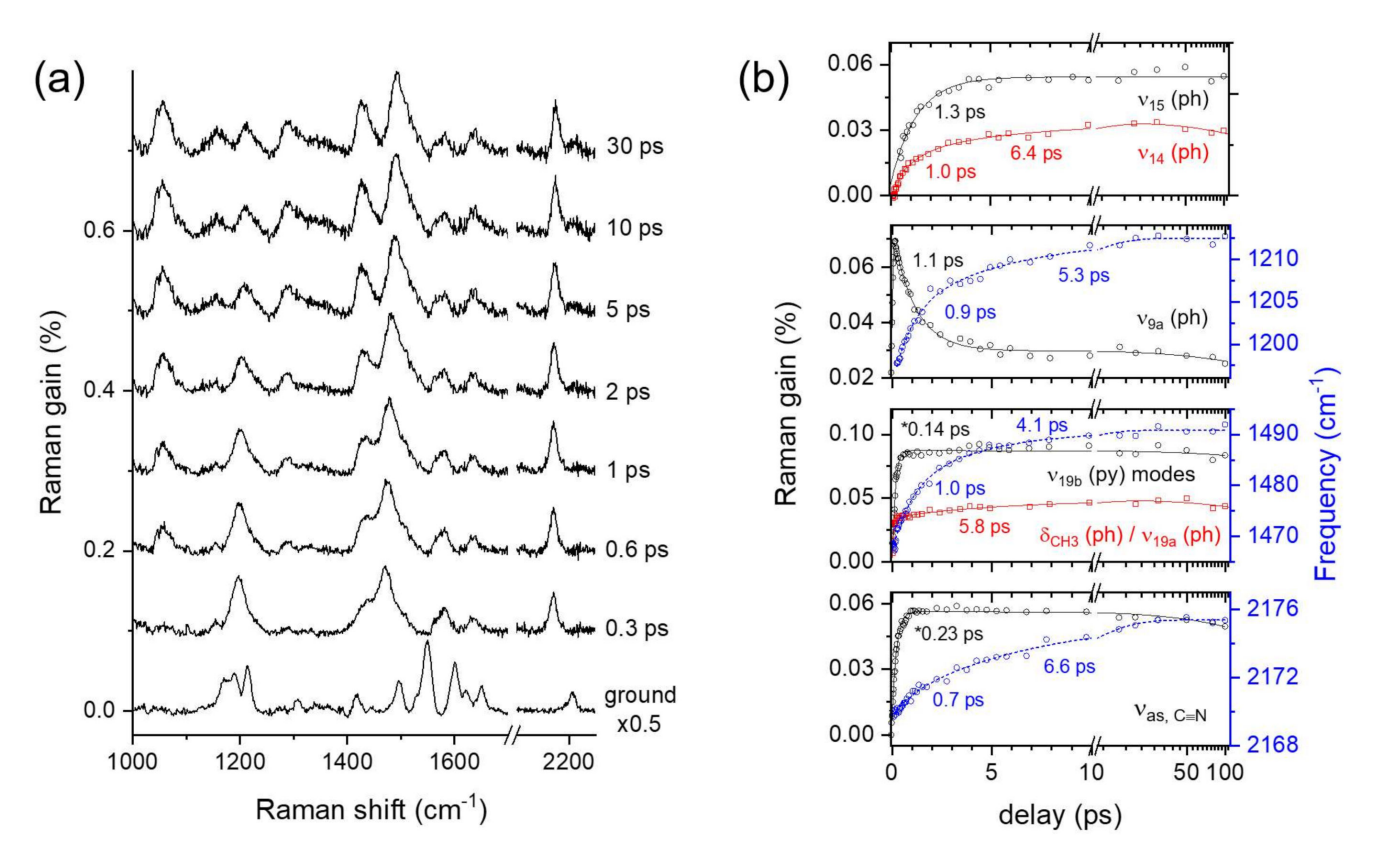
(**a**) Femtosecond stimulated Raman spectra of DCM in DMSO solution with 403 nm excitation and (**b**) the population and structural dynamics of major vibrational modes: ν_15_ (ph) and ν_14_ (ph) at 1057 and 1288 cm^−1^; ν_9a_ (ph) at 1200–1212 cm^−1^; ν_19b_ (py) modes at 1471–1492 cm^−1^ and δ_CH3_ (ph) or ν_19a_ (ph) mode at 1436–1428 cm^−1^; and ν_as,C≡N_ at 2170–2175 cm^−1^. All the kinetic traces were fit with a number of exponential functions convoluted with a Gaussian function for the instrument response function of the experiment. Solid and dotted lines represent the fit results for the population and structural dynamics, respectively. The “ph” and “py” denote the phenyl and pyran ring, respectively.

The ground and excited-state Raman spectra of DCM in DMSO obtained by FSRS are shown in Figure 2a. The excited-state Raman spectra of DCM shown as the difference from the ground state spectrum, are different from the ground state spectrum with the appearance of several excited-state Raman bands and transient changes in the band position and shape upon time delay between the actinic pump and stimulated Raman probe pulses. The details of the data analysis for the FSRS results and vibrational assignments for the excited state DCM bands based on the TDDFT simulations were summarized in the Supplementary Materials. Figure 2b shows the population dynamics and peak shifts of major vibrational modes of DCM in DMSO. The excited-state bands of DCM including ν_15_ (ph) at 1057 cm^−1^ and ν_14_ (ph) at 1288 cm^−1^ sharply grow in intensity with a 1.0–1.3 ps time constant while the intensity of other ring mode ν_9a_ (ph) at 1200–1212 cm^−1^ decreases with the very similar (1.1 ps) time constant to the growth of ν_15_ (ph) and ν_14_ (ph) modes. The spectral changes in the vibrational modes of phenyl group with the kinetics of 1.0–1.3 ps would strongly suggest a state transition in the excited state. As already observed in the transient absorption measurements, this represents the ICT process of DCM in the singlet excited state.

It is interesting to note that the several vibrational modes show large peak shifts instead of changes in the Raman intensity upon the ICT. The δ_CH3_ (ph) mode at 1428 cm^−1^ and ν_19b_ (py) + ν_19a_ (ph) at 1471 cm^−1^ showed strong blue-shifts of 10–20 cm^−1^, and ν_as,C≡N_ at 2170 cm^−1^ a smaller blue-shift of 5 cm^−1^ with the ICT. As shown in Figure 2b, the peak shifts of these bands showed 4–7 ps relaxation dynamics in addition to 1.0 ps dynamics of the ICT process. This is considered as the vibrational relaxation along the ICT potential surface of DCM and slightly different relaxation dynamics between the vibrational modes are understood as originating from the property of large and floppy molecules. Notably, the similar vibrational relaxation dynamics of 6 ps during the ICT was reported in the δ_CH_ and ν_C = C_ modes of DMABN [46].

The blue-shifts of ν_19b_ (py) modes in 1471–1492 cm^−1^ are considered as one of the clear pieces of evidence for the structural changes of DCM upon the ICT. These bands are separately assigned as the vibrational modes of DCM in the S_1_/LE and S_1_/ICT states based on the TDDFT simulations: the LE bands at 1471 cm^−1^ as δ_CH3_ (ph) + ν_19b_ (py) of planar conformer and the ICT band at 1492 cm^−1^ as ν_19b_ (py) + ν_19a_ (ph) of DMA rotated conformer. The similar peak shifts in these modes have also been observed in the time-resolved infrared measurements by Fleming and co-workers as the infrared-active mode at 1495 cm^−1^ [32,33].

The time-resolved Raman and infrared measurements on DMABN and its dimethyl derivative have shown the red-shifts of 5–10 cm^−1^ in the ν_C≡N_ mode, which is the opposite to the blue-shifts of 5 cm^−1^ observed for ν_as,C≡N_ of DCM [46,56,57]. The peak shifts in the ν_C≡N_ modes of DCM upon the ICT would be interpreted differently from the case of DMABN with one nitrile group. In the ground state, the symmetric stretching of nitrile, ν_s,C≡N_ of DCM shows a much larger Raman intensity than the asymmetric mode opposite to the excited state Raman spectra. The asymmetric mode appears as the major band in the excited states and the symmetric band appears as a weak band only in the ICT state at 2212 cm^−1^ (at 5–30 ps in Figure 2a). Since the symmetric stretching ν_s,C≡N_ was not observed in the LE spectrum, the peak shifts of the symmetric mode upon the ICT is not known. The TDDFT simulations estimated 2–3 cm^−1^ blue-shifts in both ν_C≡N_ modes with the DMA rotation, but the results with DMAP rotation showed quite unfeasible results strongly depending on the level of the DFT methods. In general, the red-shifts of ν_C≡N_ can be understood as the strong solvatochromic shifts of nitrile group with the increasing dipole moment in the ICT state [58]. Our FSRS results on the ICT of DCM in the DMSO solution shows the similar red-shifts of 15–20 cm^−1^ compared to the ground state frequencies (2190 → 2170–2175 cm^−1^) for ν_s,C≡N_ while the symmetric stretching showed the small blue-shifts of 5 cm^−1^ (2207 → 2212 cm^−1^). The blue-shift of the ν_as,C≡N_ of DCM in DMSO solution during the ICT would also be interpreted as originating from the vibrational relaxation along the anharmonic potential surface of the charge-transferred (CT) conformer. The bandwidths of ν_9a_ (ph) at 1200–1212 cm^−1^ and ν_as,C≡N_ at 2170–2175 cm^−1^ also showed a decrease with 4–6 ps time constants (see Figure S3 in the Supplementary Materials), which supports our explanation for the vibrational relaxation along the anharmonic potential surface of the S_1_/ICT state.

Lastly, another ultrafast (150–250 fs) rising components retrieved from the population dynamics of ν_19b_ (py) modes and ν_as,C≡N_ modes in Figure 2b can be interpreted as the vibrational relaxation in the LE state. A similar vibrational relaxation component of 270 fs has been observed in the 2D electronic-vibrational spectroscopy of DCM, which is also compatible with the 0.3 ps vibrational relaxation component reported for DMABN by FSRS [32,46].

**Figure 3:**
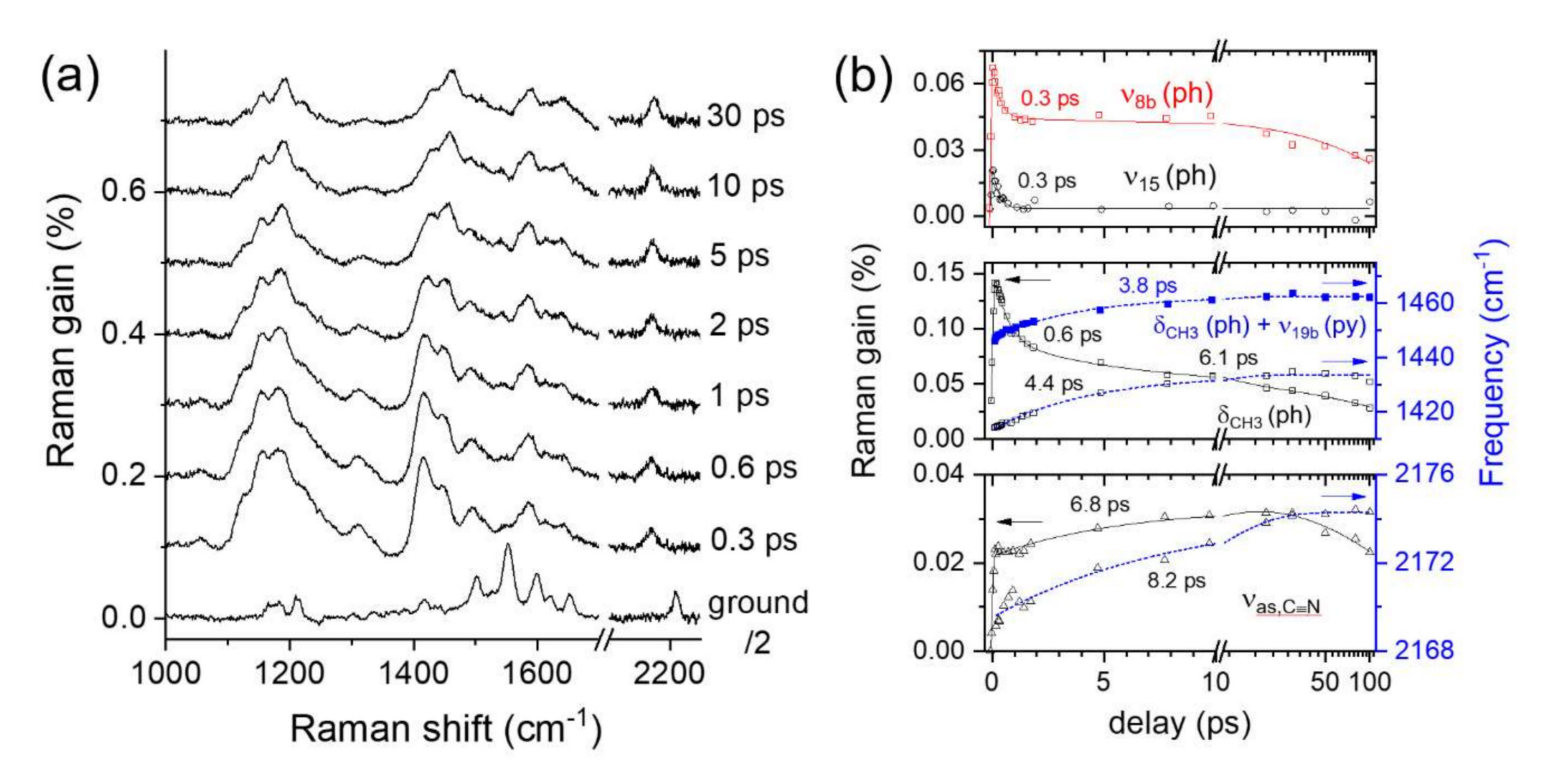
(**a**) Femtosecond stimulated Raman spectra of DCM in CHCl_3_ solution with 403 nm excitation and (**b**) the population and structural dynamics of ν_15_ (ph) at 1057 cm^−1^ and ν_8b_ (ph) at 1495 cm^−1^; δ_CH3_ (ph) at 1415–1428, δ_CH3_ (ph) + ν_19b_ (py) at 1448–1462 cm^−1^; ν_as,C≡N_ at 2171–2174 cm^−1^. All the kinetic traces were fit with a number of exponential functions convoluted with a Gaussian function for the instrument response function of the experiment. Solid and dotted lines represent the fit results for the population and structural dynamics, respectively. The “ph” and “py” denote the phenyl and pyran ring, respectively.

Figure 3a shows the time-resolved Raman spectra of DCM in the CHCl_3_ solution, which appears quite different from the LE or CT spectrum obtained with DMSO solution especially in the early time delays. However, the excited state Raman spectrum of DCM becomes very similar to the LE spectrum (at 0.3 ps in Figure 2a, for example) in 2–5 ps. Many vibrational modes are shown in Figure 3b including ν_15_ (ph) and ν_8b_ (ph) represented a fast (0.3 ps) decay. The δ_CH3_ (ph) band at 1415–1428 cm^−1^ also showed a similar decay (0.6 ps) while most of the LE vibrational bands of DCM including δ_CH3_ (ph) + ν_19b_ (py) at 1448–1462 cm^−1^ and ν_as,C≡N_ at 2171–2174 cm^−1^ appeared to be unchanged in intensity by the time delay of 5 ps or so. The vibrational relaxation in the LE vibrational bands of 4–7 ps time constants was evidenced in the population dynamics and the peak shifts as shown in Figure 3b, which is quite similar to the results of the DMSO solution.

**Figure 4:**
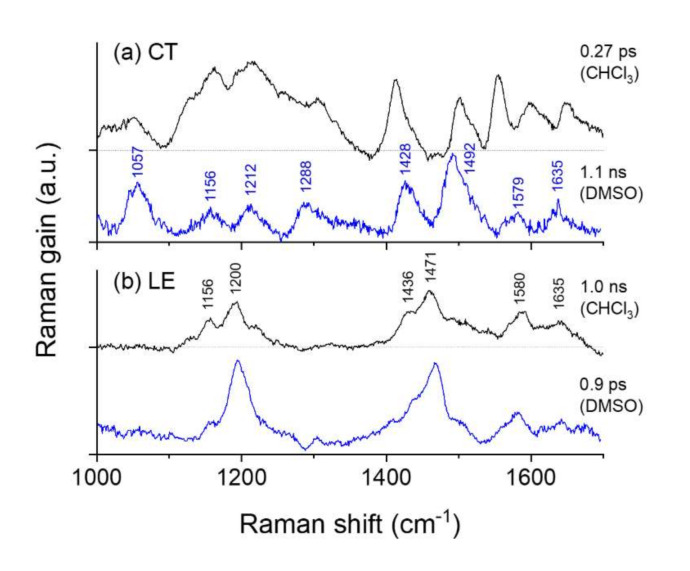
Evolution associated difference spectra (EADS) for (**a**) the intramolecular charge transfer (ICT) and (**b**) locally-excited (LE) state of DCM in CHCl_3_ and DMSO solution with the specific time constants. The kinetic information obtained from the global analysis over all the vibrational modes can be slightly different from the kinetics of a specific vibrational mode.

It is interesting to note that the early (at 0.3 ps) spectrum of DCM in CHCl_3_ can be understood as the sum of two spectral components representing the LE and ICT states of the singlet excited state. As shown in Figure S4 in the Supplementary Materials, the transient Raman spectrum of DCM in CHCl_3_ at an early (0.3 ps) time delay appeared quite similar to the scaled sums between the LE and CT spectra of DCM which were separated from the results of DMSO and CHCl_3_ solution. To further confirm the photoexcitation of both the LE and ICT states of the S_1_ state with DCM in the CHCl_3_ solution, the global analysis of the FSRS results in a wide spectral range of 800–2300 cm^−1^ were performed [55]. The details of the global analysis were summarized in the Supplementary Materials. The EADS of the fastest (0.3 ps) component obtained from CHCl_3_ results and the slowest (1.1 ns) from DMSO results were compared in Figure 4a, where almost identical spectral features representing the excited state Raman spectra of DCM in the S_1_/ICT state were observed. Similarly, the EADS of the slowest (1.0 ns) component obtained from CHCl_3_ results and the fastest (1.0 ps) from DMSO results appeared identical to each other, which is different from the CT spectra of DCM in Figure 4a, and thus represents the excited state Raman spectra of DCM in the S_1_/LE state. To summarize, both the LE and ICT state of DCM in the S_1_ excited state are photoexcited initially in CHCl_3_ solution, then the CT conformer state shows an ultrafast (0.3 ps) conversion to the LE state, which is followed by the vibrational relaxation (4–7 ps) and the population decay (1.0 ns) of the LE state. We confirm the coexistence of the LE and CT conformer states in the S_1_ excited state and the relative energy ordering between the LE and ICT states from the FSRS results of DCM. This is the first to report the structural difference between the LE and CT conformers of DCM by two distinct Raman spectra in the fingerprint frequency range, which has been numerously proposed by many experimental and theoretical studies of DCM and related push-pull type fluorophores [12,13,24,25,28,30,34]. We also showed that the ultrafast conversion (0.3 ps) from the CT to LE conformer states in weakly polar CHCl_3_ solution in addition to the conversion from the LE to ICT state (1.0 ps) in polar DMSO solution which has been numerously observed in time-resolved absorption and emission measurements [12,13,22,25,34,35]. Thus, the energy level of the CT conformer state of DCM in the S_1_ excited state appears strongly dependent on the solvent polarity, which is directly related to the formation of the ICT state and strongly red-shifted emission from the ICT state.

## 3. Discussion

### Twisted ICT State of DCM

The excited-state Raman spectra in the LE and CT conformer states of DCM were identified from the FSRS measurements in DMSO and CHCl_3_ solutions. To corroborate the experimental Raman spectra of DCM with the possible structural changes during the ICT, we performed TDDFT simulations to find the locally optimized geometries and corresponding Raman spectra. Most of the (TD)DFT studies on DCM or similar push-pull emitters have focused on the evaluation of the absorption and emission spectra which are consistent with the experimental results [27,28,29,30,59,60,61]. Investigation of the vibrational spectrum changes of a molecule during the specific excited state processes may provide important clues to further development for the DFT methods of higher accuracy. First, we estimated the potential energy curves for the ground (S_0_) and lowest singlet excited state (S_1_) along the rotational degree of freedom of DMA and DMAP groups [29,62]. The ground state geometries were optimized with the fixed dihedral angles of DMA or DMAP group at the B3LYP/6–311G(d,p) level with the polarized continuum model (PCM) for DMSO and CHCl_3_, and the vertical transition energies were calculated by the single point TDDFT simulations at each optimized ground state geometry. The S_1_ minima for both rotational angles were found with the planar geometry, but the local minima with the twisted geometry of DMA or DMAP group depending on the solvent model were also found (see Figure S7 in the Supplementary Materials).

The excited state geometry of DCM was further optimized in the S_1_ excited state with no fixed dihedral angles at the B3LYP/6–311G(d,p) and mPW1PBE/6–31G(d) levels with PCM (DMSO) starting from the optimized geometries for the “planar” minimum and two “twisted” local minima in the S_1_ potential energy surface. The optimized geometries in the S_1_ excited state including those with a twisted DMA group (dihedral angle μ = 90.0°) or DMAP (dihedral angle δ = 89.7 or 96.4°) group were obtained depending on the DFT levels, and further used for the vibrational frequency and Raman intensity simulations. Nabavi et al. recently reported that the TICT conformer along the DMAP rotation (S_1_-TransCis-δ with δ = 95.4°) may exist 380 cm^−1^ above the relaxed LE conformer in the S_1_ excited state optimized geometries of DCM in the excited state by the TDDFT simulation at the mPW1PBE/6–31G(d) level with the PCM (ethanol) [29]. Another TICT conformer along the DMA rotation (S_1_-TransCis-μ with μ = 92.0°) which exists 1260 cm^−1^ above the relaxed LE conformer was found, but the ICT to S_1_-TransCis-δ was mainly considered due to the relatively low barrier height. We updated the dihedral angles and the relative energies of two TICT conformers with the PCM model for DMSO as ΔE_S1-TransCis-μ_ = 250 (1030) cm^−1^ with μ = 90.0 (90.0)° and ΔE_S1-TransCis-δ_ = 115 (640) cm^−1^ with δ = 89.7 (96.4)° (The values outside the parentheses refer to the TDDFT results at the B3LYP/6-311G(d,p) level and those within the parentheses are the results at the mPW1PBE/6-31G(d) level.). This clearly shows the possible existence of the conformer states in the S_1_ excited state with the rotated DMA or DMAP groups. The simulation results for the excited state geometry and Raman spectrum of DCM appeared very similar between the DFT simulation levels with only minor differences (see Figure S8 and Figure S11, and Table S1 in the Supplementary Materials).

**Figure 5:**
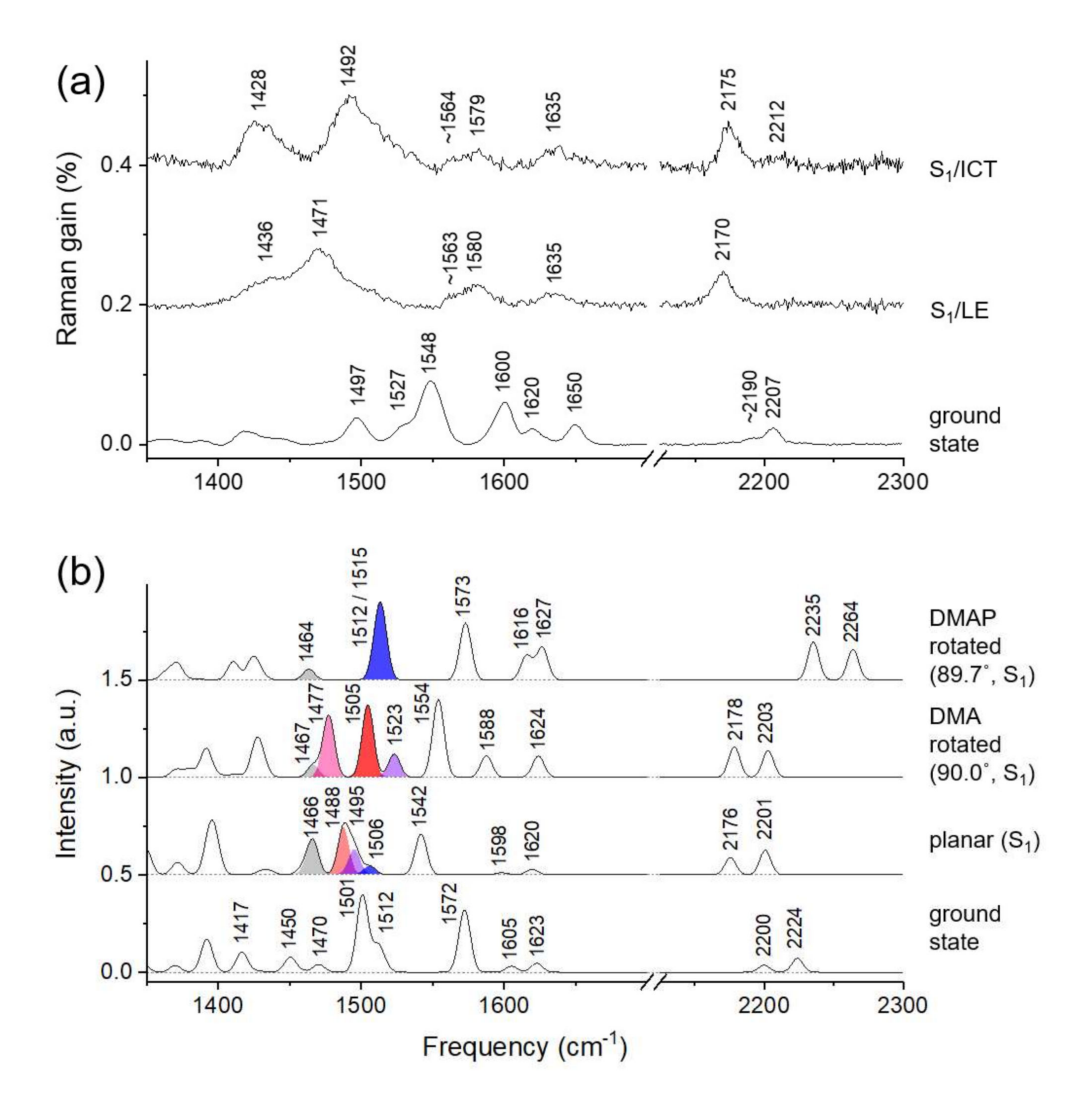
The Raman spectra of DCM in the ground state and the S_1_/LE and S_1_/ICT excited states; (**a**) the experimental results, (**b**) the TDDFT simulation results at B3LYP/6-311G(d,p) level for the optimized structures in the ground and S_1_ excited states. The spectral changes expected for ν_19a_, ν_19b_, or δ_CH3_ modes were displayed with color-codes.

Figure 5 compares the experimental Raman spectra of DCM in the ground and excited states with the simulated spectra by the (TD)DFT method at the B3LYP/6–311G(d,p) level. The excited-state Raman spectra of DCM in the S_1_/LE and S_1_/ICT states are distinct from each other and quite different from the ground state spectrum as well in many vibrational modes. First of all, the appearance of ν_15_ (ph) and ν_14_ (ph) bands at 1057 and 1288 cm^−1^, respectively, in the CT spectrum and the blue-shifts in the ν_9a_ (ph) of 1200 → 1212 cm^−1^ shown in Figure 4a, and the peak shifts of ν_as,C≡N_ (2170 → 2175 cm^−1^) upon the ICT were not clearly explained by the TDDFT simulations between the twisted geometries of the DMA or DMAP group.

The strong blue-shift in the ν_19b_ (py) + ν_19a_ (ph) mode (1471 → 1492 cm^−1^) during the ICT can also be explained by the TDDFT simulations with DMA or DMAP rotation in the excited state. The S_1_/LE vibrational bands in the 1420–1500 cm^−1^ region may consist of multiple vibrational modes considering the vibrational bands in the ground state in the 1480–1560 cm^−1^ region. We consider at least four different vibrational modes including δ_CH3_ (ph) at 1466, δ_CH3_ (ph) + ν_19b_ (py) at 1488, ν_8b_ (ph) at 1495, and ν_19a_ + δ_CH3_ (ph) at 1506 cm^−1^ shown in the simulated spectrum for the planar structure in the S_1_/LE state. The δ_CH3_ (ph) mode appears insensitive to any structural change of DMA or DMAP group, which matches to the 1436 cm^−1^ band of the S_1_/LE spectrum of DCM. The strong and broadband centered at 1471 cm^−1^ is then considered as the sum of the other three modes of δ_CH3_ (ph) + ν_19b_ (py), ν_8b_ (ph), and ν_19a_ + δ_CH3_ (ph) from the simulation results. The appearance of two strong bands at 1428 and 1492 cm^−1^ in the S_1_/ICT spectrum is better supported by the rotation of DMA rather than the DMAP group. The TDDFT simulations for the DMA rotated conformer predicted that the δ_CH3_ (ph) + ν_19b_ (py) mode of planar conformer at 1488 cm^−1^ splits into two major vibrational modes of ν_19a_ (ph) + δ_CH_ (py) at 1477 cm^−1^ and ν_19b_ (py) + ν_19a_ (ph) at 1505 cm^−1^ for the DMA rotated conformer due to the symmetry breaking, which is accordant to the experimentally observed 1428 and 1492 cm^−1^ bands in the S_1_/ICT spectrum. On the other hand, the only vibrational bands for the DMAP rotated conformer in this frequency range was ν_19a_ + δ_CH3_ (ph) at 1512/1515 cm^−1^, which is also shown at 1506 cm^−1^ for the planar conformer. However, the spectral patterns expected for the DMAP rotated conformer are different from the experimental spectrum observed in the S_1_/ICT state.

The skeletal vibrational modes appearing around 1600 cm^−1^ in the ground and excited-state Raman spectra of DCM provided the evidence for the structural changes during the ICT in the excited state. The ground state vibrational bands appearing at 1600 and 1620 cm^−1^ in Figure 5a are the symmetric and asymmetric modes of the ν_C = C_ + ν_8a_ (ph) while another band at 1650 cm^−1^ is the ν_8a_ (py). It is interesting to note that the in-plane ring stretching ν_8a_ of phenyl is strongly coupled with the central ν_C = C_ mode rather than with another in-plane stretching ν_8a_ of pyran. In the S_1_/LE spectrum, these skeletal bands appear at 1563 (sh), 1580, and 1635 cm^−1^ as shown in Figure 5a, which is assigned similarly as the symmetric and asymmetric ν_C = C_ + ν_8a_ (ph), and ν_8a_ (py) based on the TDDFT simulation for the planar structure. In the twisted conformer of the DMAP group, the strong coupling between the ν_C = C_ and ν_8a_ (ph) is no longer feasible while this coupling would still exist in the twisted conformer of the DMA group. The TDDFT results compared in Figure 5b clearly show the difference between these conformers. The ν_C = C_ mode appears at 1573 cm^−1^ and the ν_8a_ modes of phenyl and pyran ring appear at 1616 and 1627 cm^−1^, respectively. These skeletal vibrational modes experimentally observed in the S_1_/LE and S_1_/ICT states, however, showed no difference as shown in Figure 5a, which excludes the possibility of the DMAP group rotation during the ICT.

Recently, Dasgupta and co-workers have reported the FSRS of stilbazolium dye, where the twisted ICT state in the excited state was indicated by the decoupling between the ethylenic ν_C = C_ and the ν_8a_ modes of phenyl (electron donor-side) and pyridine (electron acceptor-side) rings [38]. The twist of N-methylpyridine ring and change in the molecular symmetry in the ICT state increased the Raman intensity of the ν_8a_ mode of pyridine ring at 1650 cm^−1^, which is only infrared active with the planar geometry in the ground state. We have recently obtained another piece of experimental evidence with 4-dimethylamino-4’-nitrostilbene (DMANS), which supports the twisted geometry of the DMA group for the ICT of DCM [63]. The FSRS of DMANS shown in Figure S14 in the Supplementary Materials represents an ultrafast charge transfer of ~0.3 ps in the coupled and decoupled vibrational modes of the ν_8a_ modes of two phenyls and the conjugated ν_C = C_ between them at 1575 and 1598 cm^−1^, where the twisted geometry along the nitrophenyl group was clearly explained by the TDDFT simulation results [30]. Since the transient Raman results of DCM upon the ICT show no apparent changes in the spectral region for the ethylenic ν_C = C_ and ν_8a_ modes of phenyl or pyran ring, our conclusion for the twisted ICT state of DCM as the DMA rotated conformer can further be justified by the recent results for stilbazolium dye and DMANS.

## 4. Materials and Methods 

### 4.1. Chemicals

DCM (Sigma-Aldrich, St. Louis, MO, USA) and all the solvents were used without further purification. The 0.6 mM solutions of DCM in DMSO and CHCl_3_ were recirculated by a peristaltic pump, and a 1 mm thick quartz flow cell was used for stimulated Raman measurements. For the transient absorption measurements, a dilute 50 μM solution in a 2 mm thick quartz cuvette was stirred with a magnet bar to minimize the photodamage from the excitation pulses.

### 4.2. Steady-State Absorption and Emission Measurements

Steady-state absorption spectra were measured in a UV/Vis absorption spectrometer (Scinco S3100, Scinco, Seoul, Korea) and the emission spectra were measured in a home-built time-correlated single-photon counting (TCSPC) setup based on a TCSPC module (Picoharp 300, PicoQuant, Berlin, Germany) [64,65]. Fluorescence signals from the excitation of a picosecond diode laser (P-C-405, PicoQuant) at 405 nm were filtered by a long-pass filter (Omega Optical, Brattleboro, VT, USA) at 442 nm and collected by a photomultiplier tube detector (PMA 192, PicoQuant) attached to a monochromator (Cornerstone 260, Newport, Irvine, CA, USA).

### 4.3. Transient Absorption Setup

A home-built transient absorption setup, composed of a supercontinuum white-light probe generated by sapphire crystal (3 mm thick, Thorlabs Inc., Newton, NJ, USA) and actinic pump at 403 nm generated by second-harmonic generation in a BBO crystal (θ = 29°, 0.2 mm thick, Eksma Optics, Vilnius, Lithuania) was used for transient absorption measurements [22,66]. The actinic pump was compressed in a prism pair compressor (SF10, Edmund Optics, Barrington, NJ, USA), and the probe filtered by a short-pass filter (FES700, Thorlabs Inc.) was measured in a USB spectrometer with a back-thinned CCD detector (QE65Pro, Ocean Optics, Largo, FL, USA).

### 4.4. Femtosecond Stimulated Raman Setup

A home-built FSRS setup based on a 1 kHz Ti:sapphire regenerative amplifier has been used for time-resolved Raman measurements [43,44]. The broadband Raman probe was generated by supercontinuum generation in an yttrium aluminum garnet crystal (4 mm thick, Newlight Photonics, Toronto, ON, Canada) and filtered by a long pass filter (830 DCLP, Omega Optical). The picosecond Raman pump was generated in a grating filter composed of a 1200 gr/mm grating and an f = 150 mm cylindrical lens. The pump bandwidth was adjusted by a 100 μm wide silt to obtain the spectral resolution of <8 cm^−1^. The actinic pump at 403 nm was generated by second-harmonic generation in β-barium borate crystal (θ = 29°, 0.1 mm thick; Eksma Optics) and compressed by chirped mirror pairs (Layertec GmbH, Mellingen, Germany). The Raman pump (~500 nJ/pulse) and the actinic pump (~400 nJ/pulse) were focused into the sample by f = 125 mm lenses, and the beam diameters of the Raman pump and actinic pump (~100 and ~80 μm, respectively) at the focus were adjusted slightly larger than that of Raman probe (~50 μm). The Raman probe after the sample was dispersed in a spectrograph (Triax 320, Horiba Jobin Yvon, Tokyo, Japan) and measured in a charge-coupled device (CCD) detector (PIXIS 100, Princeton Instruments, Trenton, NJ, USA). An optical chopper (MC2000, Thorlabs Inc.) was used to modulate the Raman pump at 500 Hz, and all the measurements were done in a home-built LabVIEW program.

Typically, a single Raman spectrum was acquired by averaging 10,000 probe pulses (5000 times pump on/off acquisitions each). To obtain the transient Raman spectrum of a reasonable signal to noise ratio, 30 Raman spectra were averaged for the Raman spectrum at each time delay. The Raman gain of stimulated Raman spectrum is calculated by,

(1) $$\left(\mathrm{Raman}\;\mathrm{Gain}\right)=\frac{I_{R.\mathrm{Pump}-\mathrm{ON}}-I_{\mathrm{Bkg}}}{I_{R.\mathrm{Pump}-\mathrm{OFF}}-I_{\mathrm{Bkg}}}$$

where *I*_R.Pump-ON_ and *I*_R.Pump-OFF_ represent the Raman probe intensities with and without the Raman pump, respectively, and *I*_Bkg_ is the background signal of the CCD detector.

### 4.5. Data Analysis

Transient absorption and FSRS results were analyzed by a global analysis with a sequential decay model, and a software package Glotaran was used [55,67]. The time-resolved spectra from the transient absorption and FSRS measurements can be written as a superposition of distinct kinetic components by the following relation,

(2) $$\Psi (t,\lambda )=\sum_{i=1}^{n}c_{i}(t)\varepsilon_{i}(\lambda )$$

where *c_i_*(*t*) and ε_i_(*λ*) represents the kinetics and spectra of *i*-th kinetic components. The spectra, ε_i_(*λ*) of the global analysis with a parallel decay model is often called decay-associated difference spectra (DADS), and evolution-associated difference spectra (EADS) represent the results with a sequential decay model [55].

## 5. Conclusions

Theoretical investigations on the intramolecular charge transfer (ICT) process of a laser dye DCM and related push-pull type emitters have not reached a conclusive solution for the structural identities of the highly fluorescent ICT states. In this paper, we have obtained two distinct Raman spectra for the locally-excited (LE) and charge-transferred (CT) conformers of DCM by femtosecond stimulated Raman spectroscopy, and the internal conversion dynamics between two conformers and the subsequent vibrational relaxation in the LE or CT potential surface were evidenced in numerous vibrational modes of DCM as the changes in the Raman intensity, peak position, and bandwidth. To corroborate the experimental Raman spectra with the structural change upon the ICT, we applied the time-dependent density functional theory (TDDFT) simulations for the optimized geometries and Raman spectra in the ground and excited states. Although the TDDFT simulations for the twisted ICT conformers along the dimethylamino (DMA) or dimethylaminophenyl (DMAP) rotation would not provide an unambiguous answer for the spectral identity of DCM in the S_1_/ICT state, the major spectral changes in the ν_19b_ (py) + ν_19a_ (ph) mode at 1492 cm^−1^ and no apparent changes in the spectral region of the ν_8a_ and ethylenic ν_C = C_ upon the ICT were better supported by the rotation of DMA group. Thus, we propose that the twisted ICT geometry along the DMA group would be favored for the S_1_/ICT state of DCM by now. We acknowledge that this may be incompatible with the previously reported theoretical results. Nonetheless, we hope that these experimental Raman results on push-pull emitters can stimulate further theoretical efforts on the molecular geometries and vibrational spectra in the excited states, and further applications using the sensitizers with the charge transfer.

## Supplementary Materials

Supplementary materials can be found at https://www.mdpi.com/1422-0067/21/21/7999/s1.

## Abbreviations

DCM	4-dicyanomethylene-2-methyl-6-(p-dimethylaminostyryl)-4H-pyran
LE	locally-excited
ICT	intramolecular charge transfer
DMA	dimethylamino
DMAP	dimethylaminophenyl
TICT	“twisted” ICT
PICT	“planar” ICT
CS INDO	conformation spectra-intermediate neglect of differential overlap
MRCI	multi references configuration interaction
CASSCF	complete active space self-consistent-field
DFT	density functional theory
TDDFT	time-dependent density functional theory
CT	charge-transferred
DMSO	dimethylsulfoxide
FSRS	femtosecond stimulated Raman spectroscopy
DMABN	4-(dimethylamino)benzonitrile
CHCl_3_	chloroform
EADS	evolution associated difference spectra
ph	phenyl
py	pyran
ps	picosecond
fs	femtosecond
PCM	polarized continuum model
CCD	charge-coupled device
