# Supplementary material for: Twisted Intramolecular Charge Transfer State of a “Push-Pull” Emitter
Source: Int J Mol Sci. 2020 Oct 27;21(21):7999. doi: 10.3390/ijms21217999 (PMC7662227; doi:10.3390/ijms21217999)
Supplement: Supplementary file 1 [file ijms-21-07999-s001.pdf]

[Supplementary Materials]

Twisted intramolecular charge transfer state of a "push-pull" emitter

*Sebok Lee, Myungsam Jen, and Yoonsoo Pang**

Department of Chemistry, Gwangju Institute of Science and Technology, 123

Cheomdangwagi-ro, Buk-gu, Gwangju 61005, Republic of Korea

*E-mail: ypang@gist.ac.kr

Table of Contents

1. Transient absorption measurements 2
   Excited state dynamics of DCM in DMSO 2
   Excited state dynamics of DCM in $CHCl_3$ 2
2. Femtosecond Stimulated Raman measurements 5
   Global analysis of FSRS results 10
3. DFT simulation for the excited state geometry and Raman spectra 12
   Computational Details 12
   Vibrational assignments of DCM in the ground state 17
   Vibrational assignments of DCM in the excited state 22
4. FSRS of 4-dimethylamino-4′-nitrostilbene 29
5. References 30

1. Transient absorption measurements

Excited state dynamics of DCM in DMSO

The transient absorption spectra of DCM in the DMSO solution with 403 nm excitation were shown in Figure S1(a). In an early time delay (1 ps for example), the excited state absorption (ESA) band at 475 and 540 nm (shoulder), and the stimulated emission (SE) centered at 630 nm appeared. The ESA band at 475 nm showed a blue-shift to 465 nm, and the SE band a red-shift to 658 nm in 10 ps. These peak shifts in the ESA and SE bands of DCM represent the internal conversion to the intramolecular charge transfer (ICT) state and the subsequent vibrational relaxation as already reported in many previous studies.[3] Once the population reaches the bottom of the potential surface of the ICT state, the transient absorption signal only shows a decrease in intensity without any major spectral change. The lifetime of the charge transfer state was obtained as 2.3 ns in our measurements. The global analysis with a sequential decay model gave the best fit to the experimental results, and three lifetimes of 0.9 ps, 3.6 ps, and 2.1 ns were resolved. The evolution-associated difference spectra (EADS) of the global analysis were shown in Figure 1(b), where the 0.9 ps component represents the locally-excited (LE) population in the S_1 state. The 3.6 ps component is assigned as the vibrational relaxation of the charge-transferred (CT) state, and the longest (2.1 ns) component as the lifetime of the CT state.

Excited state dynamics of DCM in $CHCl_3$

The transient absorption spectra of DCM in $CHCl_3$ solution with 403 nm excitation shown in Figure S1(b) showed the ESA band in the range of 430-520 nm and the SE band in the range of 530-750 nm. The emission bands appearing as two bands at 572 and 635 nm in an early time delay of 0.5 ps were changed into a single emission band at 600 nm with a smaller bandwidth by the 2-10 ps delay and later. The ESA band also showed small spectral changes in 2-10 ps; the band became slightly broader with the appearance of a shoulder band at 525 nm. The ICT

formation was not observed in the $CHCl_3$ solution, and the vibrational relaxation of DCM in the LE state was reported as ~7 ps.[8] Similarly, the global analysis with a sequential decay model resolved three kinetic components of 390 fs, 4.0 ps, and 620 ps as the corresponding EADS were shown in Figure 1(c). The 4.0 ps and 620 ps components are considered as the vibrational relaxation and the lifetime of the LE state, respectively. However, the identity of the fastest (390 fs) component is not clear considering only the transient absorption results. As shown in the femtosecond stimulated Raman spectroscopy (FSRS) results of DCM in the $CHCl_3$ solution (shown in Figure 3), the 390 fs component is assigned as a small contribution from the CT conformer of DCM in the S_1 state. Two SE bands appearing in the early time delays may represent the co-excitation of the LE and CT states of DCM in the $CHCl_3$ solution. As clearly shown later in the FSRS results, the CT population immediately converts to the LE state with a time constant of 300 fs.

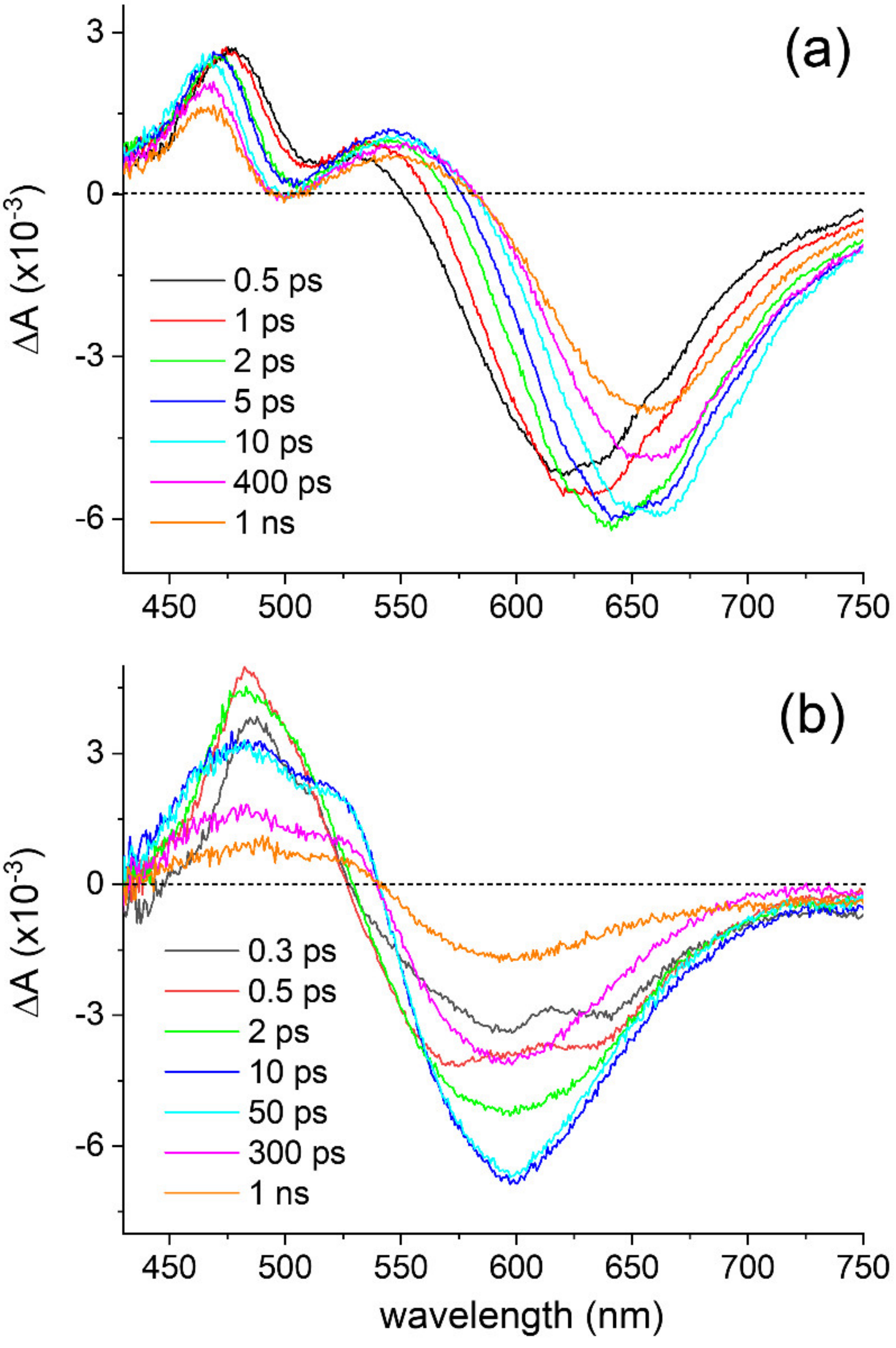

Figure S1. Transient absorption spectra of DCM in (a) DMSO and (b) $CHCl_3$ solution obtained with the 403 nm excitation.

2. Femtosecond Stimulated Raman measurements

Figure S2 shows the time-resolved stimulated Raman spectra of DCM in DMSO and $CHCl_3$ solution with 403 nm excitation. The ground state spectrum of DCM in each solution was subtracted from the transient Raman spectra at time delays between -1 and 100 ps. The background signal beneath all the Raman bands may represent the tail of the strong transient absorption signals in the probe wavelength of 850-950 nm. The increased Raman gains of the transient absorption signal represent the contribution from the SE. However, the SE background signals were perfectly removed in a low order polynomial fit. The difference stimulated Raman spectra of DCM after the polynomial fit were shown in Figure 2(a) and Figure 3(a). The amplitude, frequency, and bandwidth of each Raman band were obtained from the spectral fit with multiple Gaussian functions. The representative population changes and peak-shifts for the major vibrational bands of DCM in DMSO and $CHCl_3$ solution were summarized in Figure 2(b) and 3(b), respectively. Besides, the changes of bandwidth for the ν_{9a} (ph) at 1200 cm^{-1} and the $\nu_{as,C\equiv N}$ at 2170 cm^{-1} shown in Figure S3 also showed the time constant of 3-5 ps for the vibrational relaxation in the CT state of DCM in DMSO. All the population dynamics, frequency shifts, and bandwidth changes were fit with several exponential functions convoluted by a Gaussian representing the instrumental response function (IRF) of the experiment.

Two distinct Raman spectra of the LE and CT conformers of DCM in the S_1 excited state were obtained from the FSRS measurements in DMSO (polar) and $CHCl_3$ (less polar) solution. In the DMSO solution, the Raman spectra at early time delays (0.3 ps, for example, for LE state) and the later time delays (30 ps, for example, for CT state) are distinct from each other as shown in Figure 2(a). In the $CHCl_3$ solution, the Raman spectra at early time delays are somewhat complicated but they become very similar to the LE spectrum (observed at 0.3 ps in DMSO solution) at later time delays (30 ps, for example). Then the early delay (0.3 ps, for

example) Raman spectra of DCM in $CHCl_3$ solution can be understood as the sum of the spectral components for the LE and CT states.

The excited state spectrum of DCM in the LE and CT conformer state can be separately observed in the DMSO and $CHCl_3$ solutions. The early delay Raman spectrum of DCM in DMSO and the later delay spectrum in $CHCl_3$ are considered as the LE conformer spectrum. The later delay spectrum in DMSO is considered as the CT conformer spectrum. In Figure S4, the Raman spectrum of DCM in $CHCl_3$ at 0.3 ps was compared with the 2:1 scaled sum between the 0.3 ps (LE) and 30 ps (CT) spectrum in DMSO, and another 2:1 scaled sum between the 30 ps (LE) spectrum in $CHCl_3$ and the 30 ps (CT) spectrum in DMSO. The major peak positions in the Raman spectrum of DCM in $CHCl_3$ at 0.3 ps were very similar to two spectra composed of the LE and CT conformer spectra obtained from the DMSO and $CHCl_3$ solution. The Raman bands of DCM at 1057, 1312, 1425, and 1495 cm^{-1} in Figure S4(a) show a single exponential decay of 300 fs time constant. From this, we confirm that two distinct Raman spectra of DCM were measured in the $CHCl_3$ solution and that the CT conformer which is excited simultaneously with the LE conformer converts rapidly to the LE conformer.

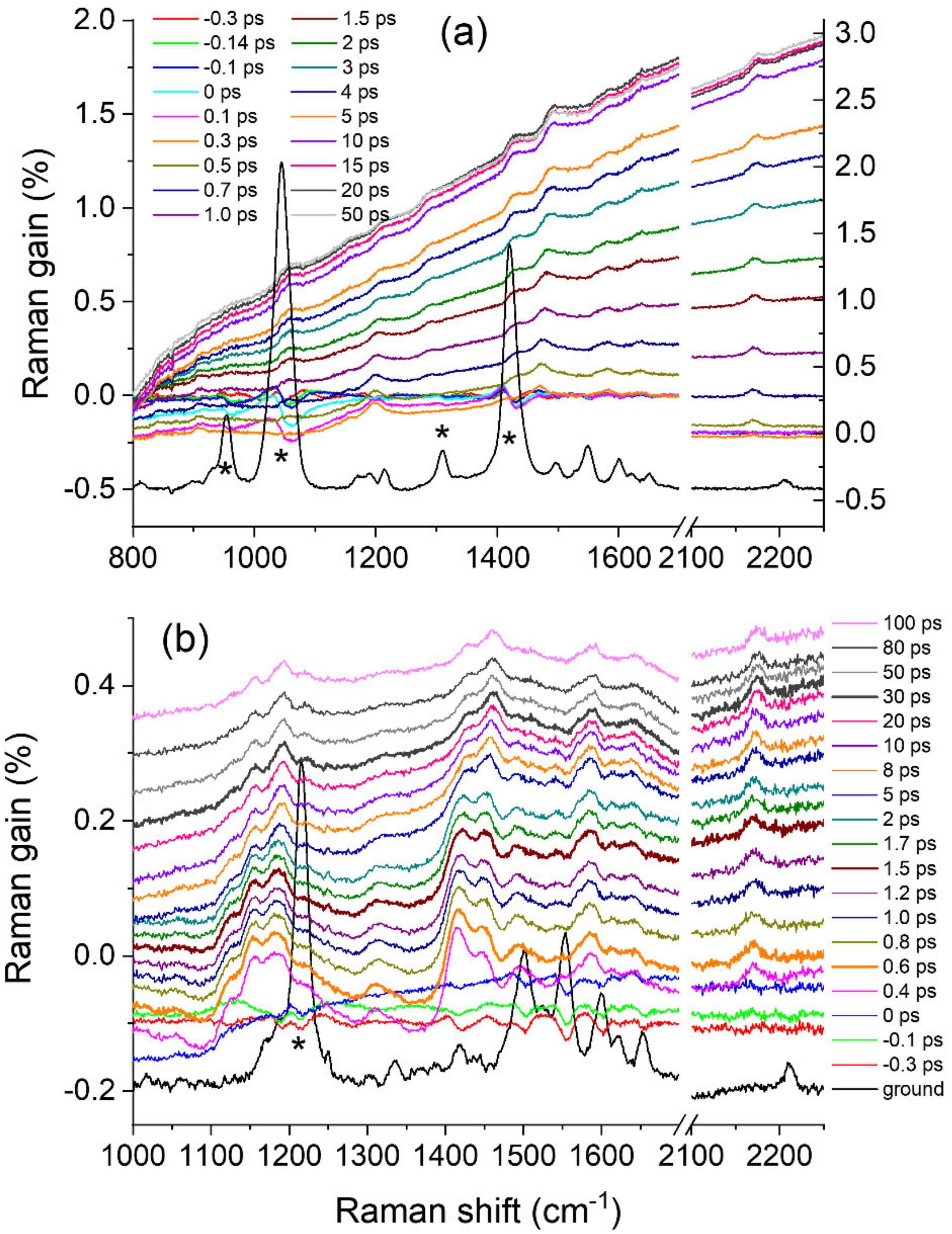

Figure S2. Difference stimulated Raman spectra of DCM in (a) DMSO and (b) $CHCl_3$ solution following the 403 nm photoexcitation. The solvent vibrational bands in the ground state spectra were marked by an asterisk (*).

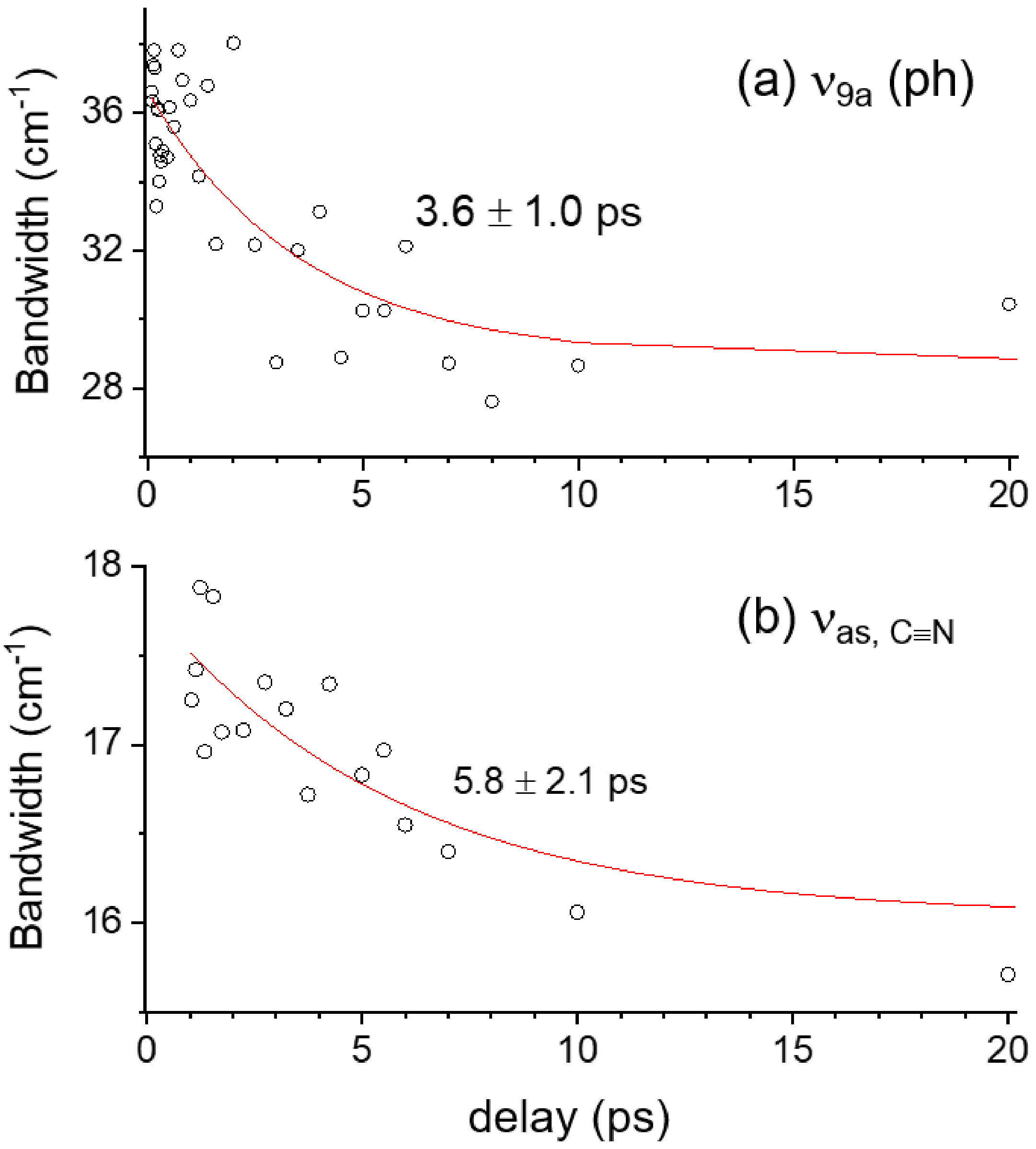

Figure S3. The structural dynamics of DCM in DMSO; (a) ν_{9a} (ph) at 1200 cm^{-1} and (b) $\nu_{as, C\equiv N}$ at 2170 cm^{-1}.

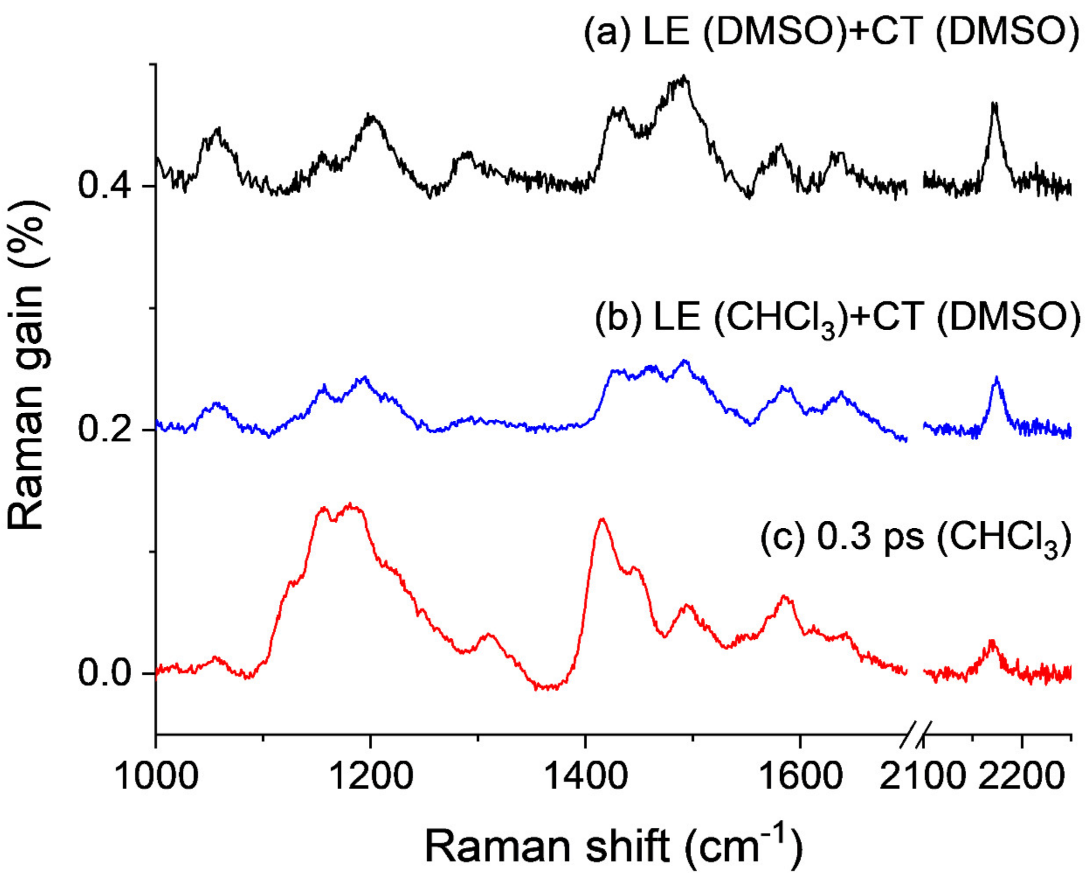

Figure S4. Comparison of transient stimulated Raman spectra; (a) LE spectrum in DMSO (0.3 ps) + CT spectrum in DMSO (30 ps), (b) LE spectrum in $CHCl_3$ (30 ps) + CT spectrum in DMSO (30 ps), and (c) DCM spectrum in $CHCl_3$ (0.3 ps). These results show that the DCM spectrum in $CHCl_3$ at early time delays originate from both the LE and CT conformers in the S_1 excited state.

Global analysis of FSRS results

The global analysis with the sequential model was applied to the FSRS results of DCM in DMSO and $CHCl_3$ solution. Three kinetic components of 0.9 ps, 4.3 ps, and 1.1 ns were obtained for the FSRS results of DCM in the DMSO solution, and the corresponding EADS were shown in Figure S5(a). The EADS of 0.9 ps component shows clear differences in many vibrational bands from the spectrum of 4.3 ps component. The 0.9 ps component which is directly populated by the actinic pump, represents the LE conformer of DCM. The EADS of 4.3 ps and much longer 1.1 ns components shares many major vibrational bans with slight peak shifts. Thus the 4.3 ps component is assigned as the vibrational relaxation in the potential surface of the CT conformer and the 1.1 ns component represents the CT conformer of DCM. All three components of DCM obtained from the FSRS in DMSO are similar to those obtained from the transient absorption measurements.

Three kinetic components of 270 fs, 5.6 ps, and 1.0 ns were obtained from the FSRS of DCM in the $CHCl_3$ solution. The EADS of the three components are shown in Figure S5(b). The EADS of 270 fs component which represents the CT conformer of DCM, is very similar to the EADS of 1.1 ns component in DMSO solution, but small differences in several vibration bands still exist. The CT conformer was populated together with the LE conformer, and rapidly converts to the LE conformer, as clearly shown in Figure 3(b) and Figure 4. A more thorough global analysis with a branching and recombining dynamics between the CT and LE conformer of DCM would be needed. The 5.6 ps and 1.0 ns components in the dynamics of DCM in $CHCl_3$ are very similar to each other as the two components of the CT conformers showed the 4.3 ps and 1.1 ns components in the DMSO solution. Therefore, the 5.6 ps component is considered as the vibrational relaxation of the LE state, and the 1.0 ns is attributed to the lifetime of the LE state.

Two distinct excited state Raman spectra of DCM which represent the LE and CT conformer states are well represented by the EADS of DCM in DMSO and $CHCl_3$ solution. Figure 4(a) compares the EADS for the 270 fs component of $CHCl_3$ results and the 1.1 ns component of DMSO results for the CT conformer of DCM in the S_1 state. Figure 4(b) compares the EADS for the 1.0 ns component of $CHCl_3$ results and the 0.9 ps component of DMSO results for the LE conformer of DCM in the S_1 state.

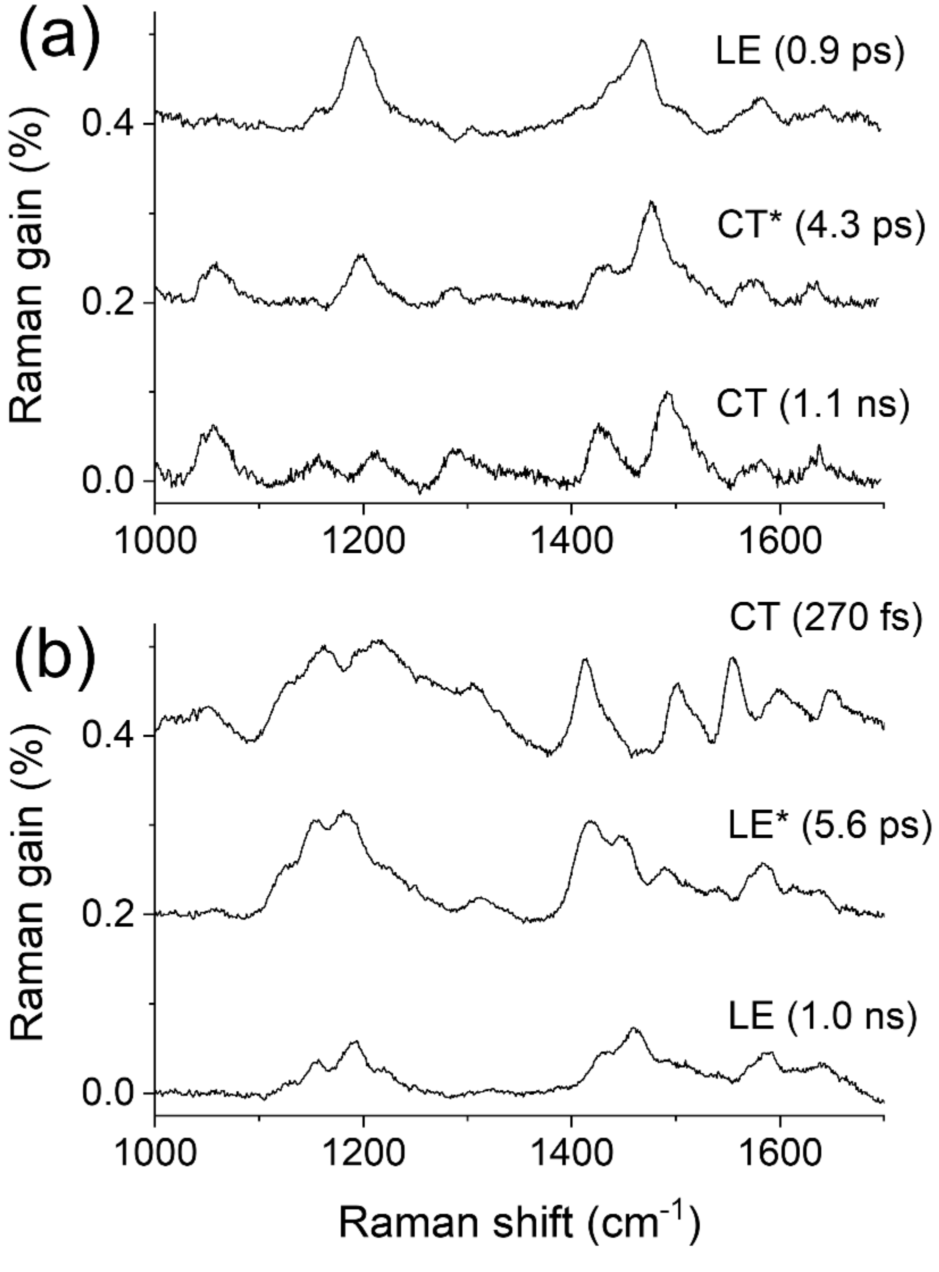

Figure S5. EADS for the FSRS of DCM in (a) DMSO and (b) $CHCl_3$ solution

3. DFT simulation for the excited state geometry and Raman spectra

Computational Details

The optimized structure and the Raman spectrum of DCM were simulated by a density functional theory (DFT) method, and the Gaussian 09 software was used.[9] The structure of DCM and key notations for the dihedral rotations and normal mode vibrations are visualized in Figure S6. The ph and py refer to the phenyl and pyran ring, respectively, the C_a, C_b, C_C, and C_d notations were used to represent the skeletal vibrational modes of DCM.

The molecular geometry of DCM in the ground state was optimized by using the B3LYP exchange-correlation functional and 6-311G(d,p) basis set with the polarizable continuum model (PCM) for solvent effects. The potential energy surfaces of DCM in the gas phase, and in the $CHCl_3$ and DMSO solution along the dihedral degree of freedoms for dimethylamino (DMA; μ) and dimethylaminophenyl (DMAP; δ) group rotation were estimated in the ground and excited singlet states.[10] First, the molecular structures of DCM in the ground state with a fixed dihedral angle at a specific value. The potential energy curves in the ground state shown

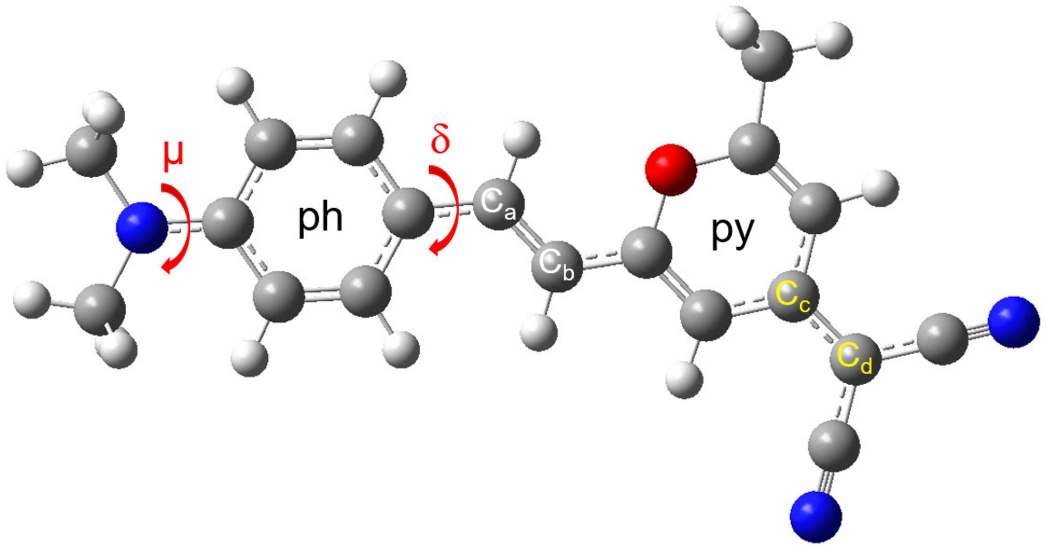

Figure S6. Molecular geometry of DCM. The ph and py refer to the phenyl and pyran ring, respectively, the C_a, C_b, C_C, and C_d notations were used to represent the skeletal vibrational modes of DCM.

in Figure S7 were obtained by comparing the relative energy for the optimized structure at each dihedral angle. The planar molecular geometry was preferred for both dihedral angle rotations in all conditions, and the energy difference of the twisted molecular structures (δ or μ = 90°) relative to the ground planar geometry was estimated as 0.4-0.6 eV (40-60 kJ/mol). Then, the vertical transition energies were calculated in a single point time-dependent (TD) DFT simulation at the B3LYP/6-311G(d,p) level with the PCM level by using each optimized structure with a specific dihedral angle μ and δ. The potential energy curves of DCM in two lowest excited states, S_1 and S_2 based on the single point TDDFT simulations in the gas phase, and in the $CHCl_3$ and DMSO solution were added in Figure S7. A local minimum for the twisted (μ = 90°) DMA group was found in the S_1 excited state regardless of the conditions; the twisted geometry was located at about the same level as the S_1 planar geometry in the gas phase, but 0.2-0.3 eV above than the planar geometry in the solution conditions. However, for the DMAP group rotation, the local minimum was located for the twisted (δ = 90°) geometry only in the gas phase. The twisted geometry in the gas phase was located at about the same level as the S_1 planar geometry, and 0.2 eV higher than the planar geometry in the solution conditions. The geometry optimization of DCM in the S_1 excited state with no fixed degree of freedom was further performed by using the twisted (μ or δ = 90°) molecular geometry from the potential energy calculations as the starting point. The optimized molecular structures of DCM with the "twisted" DMA (μ = 90.0° for B3LYP/6-311G(d,p) and μ = 90.0° for mPW1PBE/6-31G(d)) or DMAP group (δ = 89.7° for B3LYP/6-311G(d,p) and δ = 96.4° for mPW1PBE/6-31G(d)) were obtained for the S_1 CT state, which is considered compatible to the previous reports by TDDFT methods.[10-11] The optimized structures of DCM in the S_1 excited state by using the B3LYP and mPW1PBE correlation functionals are summarized in Figure S8 and Table S1.

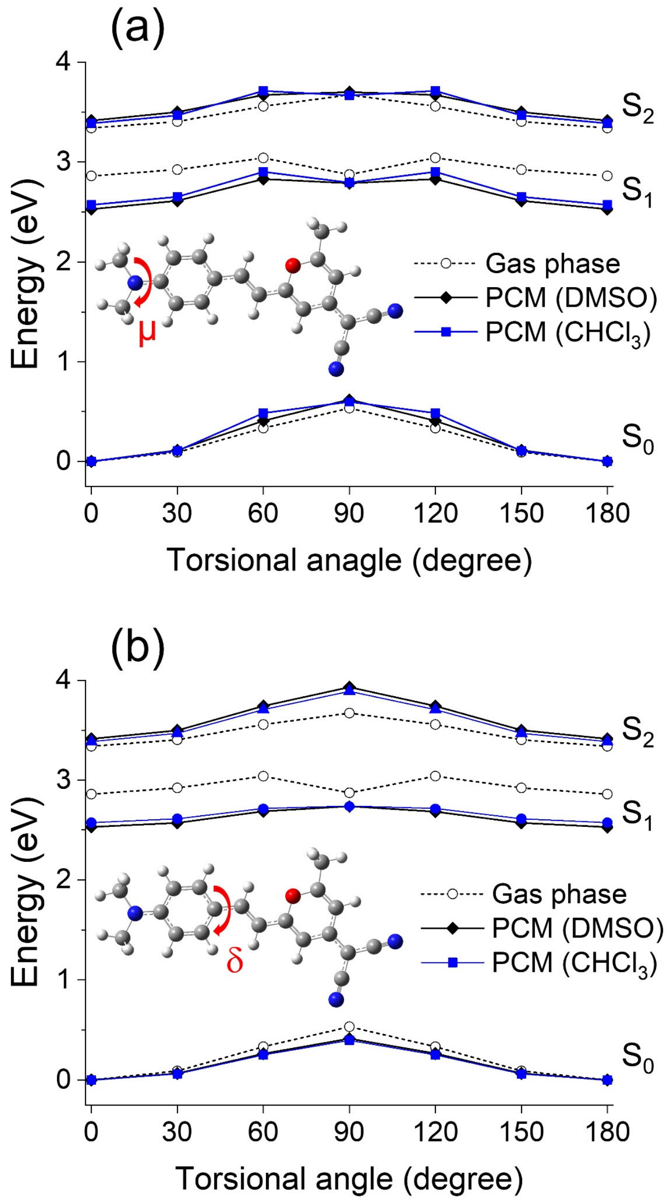

Figure S7. Potential energy curve of DCM in the gas phase and in DMSO and $CHCl_3$ solution based on the ground state optimized geometry and the single point TDDFT simulations at the TD-B3LYP/6-311G(d,p) level; the dihedral rotations along (a) the dimethylamino (DMA) and (b) dimethylaminophenyl (DMAP) group were drawn separately.

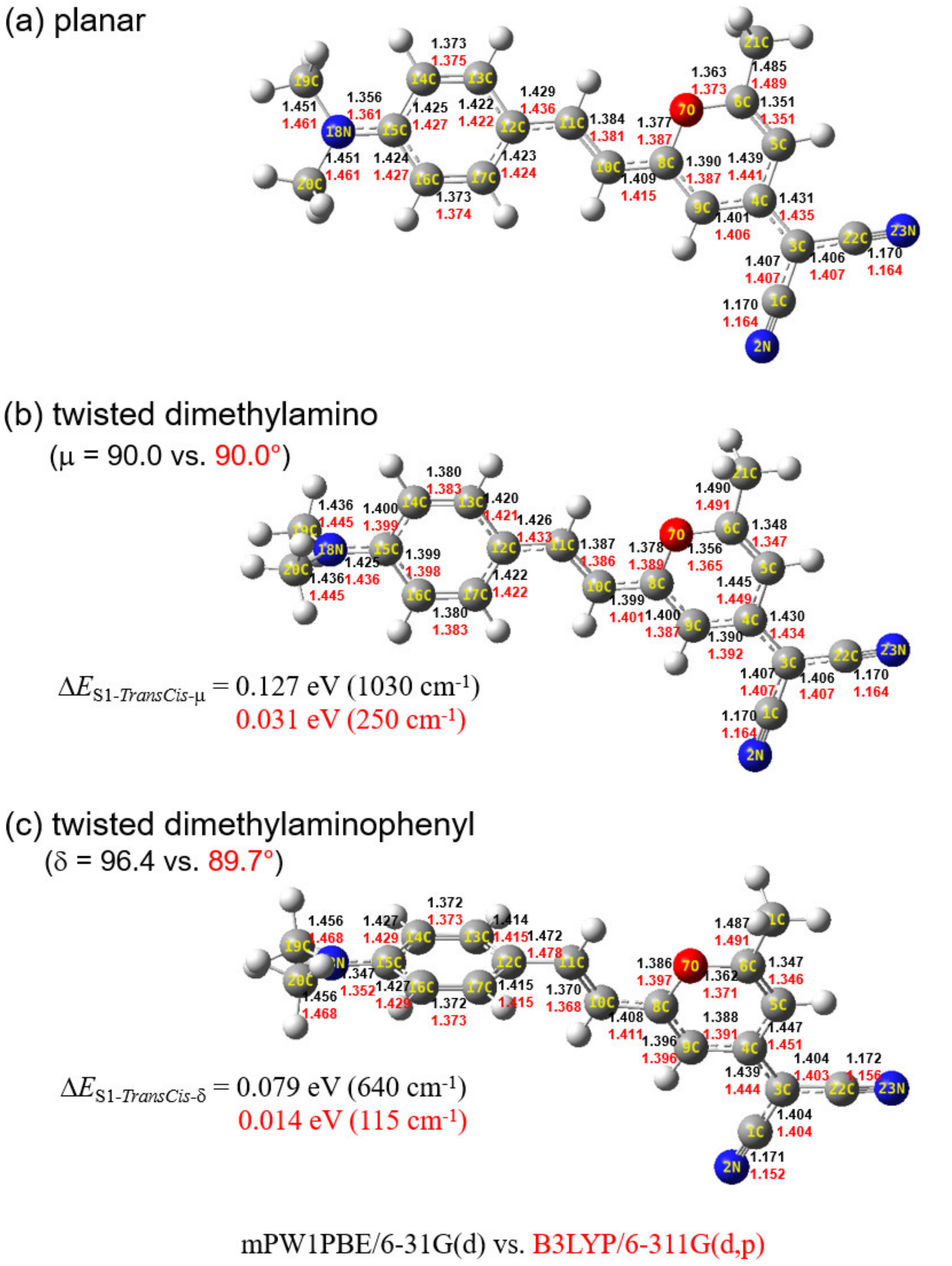

Figure S8. The optimized structures of DCM in the S_1 excited state with (a) planar, (b) twisted dimethylamino (DMA) group, and (c) twisted dimethylaminophenyl (DMAP) group. The TDDFT simulations at the B3LYP/6-311G(d,p) and mPW1PBE/6-31G(d) levels were compared with the energy different from the S_1 minima.

Table S1. The optimized structure of DCM in the S_1 excited state with the "twisted" dimethylamino (DMA) and dimethylaminophenyl (DMAP) groups

degree of freedom	B3LYP/6-311G(d,p)	mPW1PBE/6-31G(d)	
	DMSO	DMSO	EtOH[a]
1. dimethylamino (DMA)			
**bond angle (°)**			
C19-N18-C20	121.9	121.7	121.7
C14-C15-C16	120.8	120.9	120.9
C19-N18-C15	119.1	119.2	119.2
N18-C15-C14	119.6	119.5	119.5
**dihedral angle μ (°)**			
C19-N18-C15-C16	89.7	87.8	87.8
C19-N18-C15-C14	90.3	92.2	92.2
C20-N18-C15-C16	90.3	91.8	91.8
C20-N18-C15-C14	89.7	88.2	88.2
2. dimethylaminophenyl (DMAP)			
**bond angle (°)**			
C13-C12-C17	117.7	117.6	117.6
C12-C11-C10	122.8	122.4	122.3
C17-C12-C11	121.2	121.0	121.0
**dihedral angle δ (°)**			
C17-C12-C11-C10	89.7	96.4	95.4

[a] from Ref. [11]

[b] The rotation angle (μ) of the DMA group is evaluated as the average of two dihedral angles, ∠(C19-N18-C15-C16) and ∠(C19-N18-C15-C14), or ∠(C20-N18-C15-C16) and ∠(C20-N18-C15-C14).

Vibrational assignments of DCM in the ground state

The vibrational assignments for DCM in the ground state were based on the optimized geometry by the DFT simulation at the B3LYP/6-311G(d,p) level with PCM (DMSO) shown in Figure S8(a). The Raman frequencies and intensities for the vibrational normal modes of DCM obtained from the DFT simulation at the B3LYP/6-311G(d,p) level were compared with the experimental Raman spectrum in Figure S9. The simulated vibrational frequencies of DCM with the scaling factor of 0.967 for the B3LYP/6-311G(d,p) level[12-13] appears quite different from the experimental results as compared in Figure S9(a) and (b). However, we found that the simulated vibrational frequencies of DCM from the DFT method become very similar to the experimental results except the $\nu_{C\equiv N}$ modes at 2190 (sh)[i] and 2207 cm^{-1} when a slightly larger scaling factor of 0.984 was applied as shown in Figure S9(c). The vibrational assignments for the major vibrational normal modes of DCM were summarized in Table S2 and also displayed in Figure S10.

The vibrational modes appearing at 1170, 1190, and 1214 cm^{-1} in the ground spectrum of DCM in DMSO are matched to the ν_{9a} + δ_{CH3} (ph), ν_{9a} + δ_{CH3} (ph) + $\nu_{C\text{-}CH3}$ (py), and ν_{9a} (ph) + δ_{CH} (py) modes, respectively. The vibrational bands in the region of 1250-1450 cm^{-1} are also well matched to the simulated results, and most of these modes are related to the δ_{CH3} and δ_{CH} modes. The strong bands at 1497, 1527 (sh), 1548, 1600, 1620, 1650 cm^{-1} in the ground spectrum of DCM in DMSO are assigned as the δ_{CH3} (ph), ν_{19b} (py) + δ_{CH3} (ph), ν_{19a} + δ_{CH3} (ph), $\nu(C_a{=}C_b)$ + ν_{8a} (ph) (symmetric), $\nu(C_a{=}C_b)$ + ν_{8a} (ph) (asymmetric), and ν_{8a} (py), respectively. The separate bands appearing at 2190 (sh) and 2207 cm^{-1} in the ground spectrum of DCM are unambiguously assigned as the $\nu_{as,C\equiv N}$ and $\nu_{s,C\equiv N}$, respectively.

---

[i] shoulder band

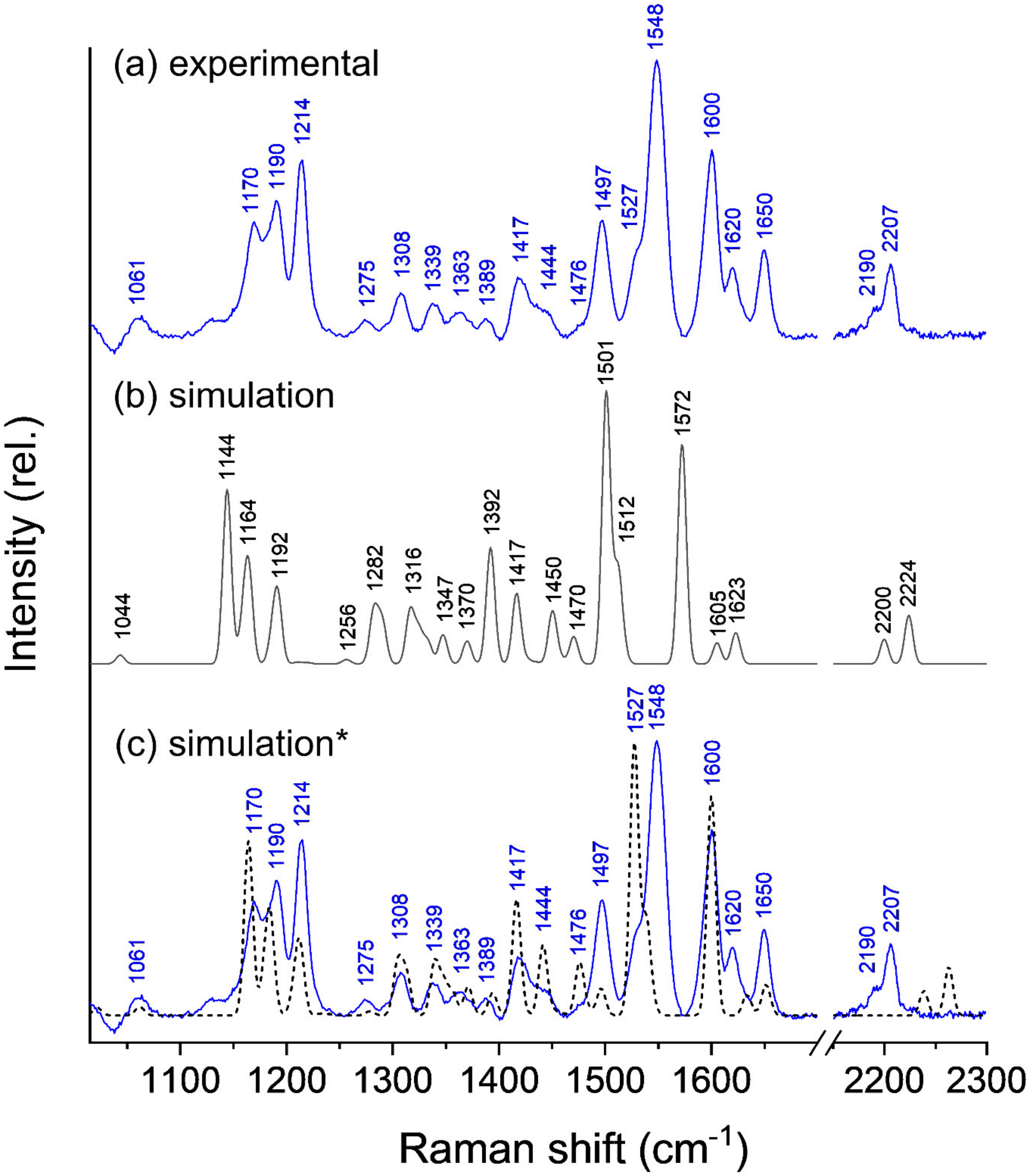
(a) experimental
1061
1170
1190
1214
1275
1308
1339
1363
1389
1417
1444
1476
1497
1527
1548
1600
1620
1650
2190
2207
(b) simulation
1044
1144
1164
1192
1256
1282
1316
1347
1370
1392
1417
1450
1470
1501
1512
1572
1605
1623
2200
2224
(c) simulation*
1061
1170
1190
1214
1275
1308
1339
1363
1389
1417
1444
1476
1497
1527
1548
1600
1620
1650
2190
2207
Intensity (rel.)
1100
1200
1300
1400
1500
1600
2200
2300
Raman shift (cm-1)

Figure S9. Ground state Raman spectra of DCM; (a) stimulated Raman spectrum in DMSO solution, (b) simulated spectrum by the DFT method at B3LYP/6-311G(d,p) level (with a scaling factor of 0.967) with PCM (DMSO). The experimental spectrum appears very similar to (c) the simulated spectrum rescaled with a slightly larger scaling factor of 0.984.

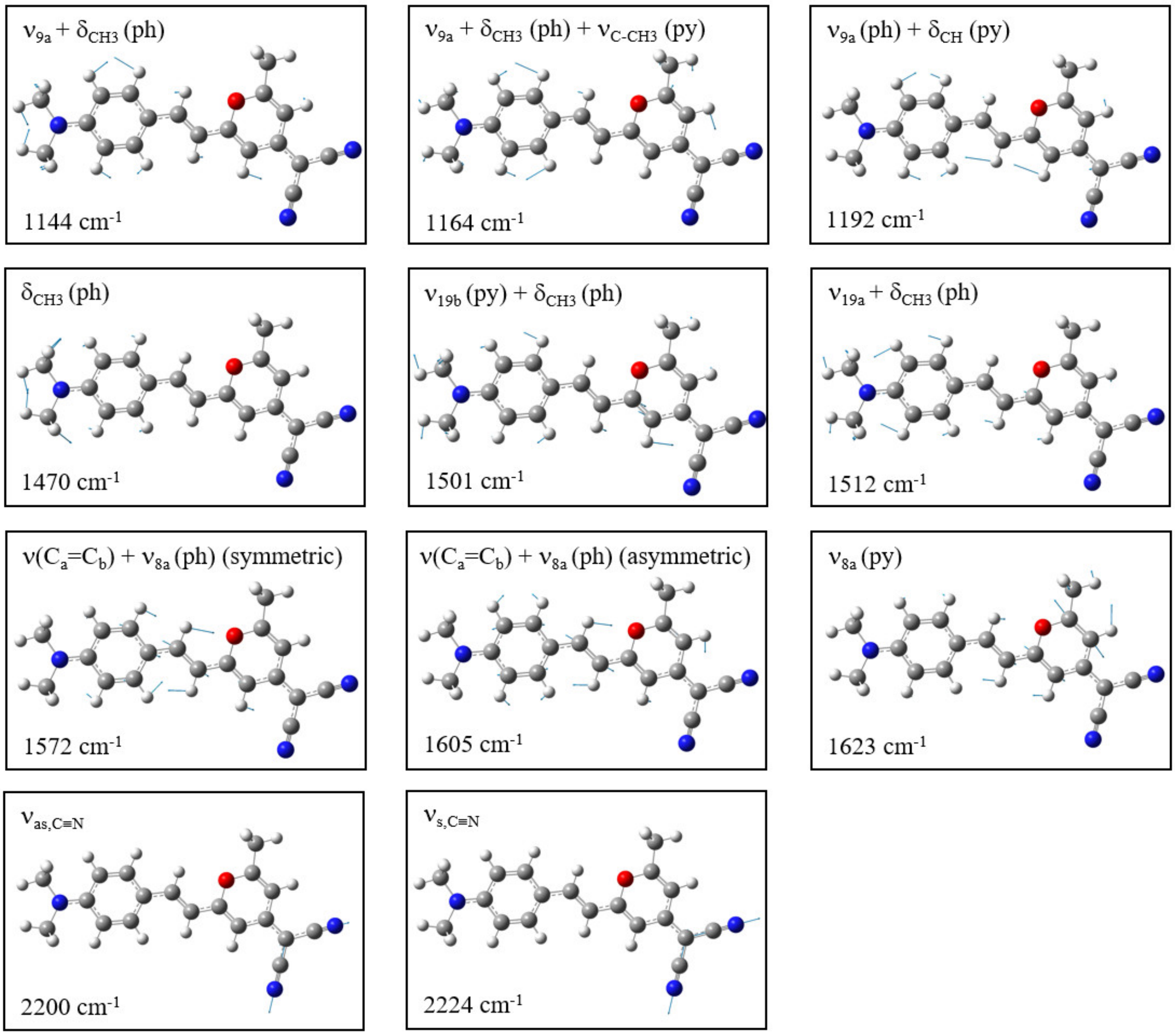

Figure S10. Vibrational normal modes of DCM in the ground state obtained from the DFT simulation at the B3LYP/6-311G(d,p) level with PCM (DMSO). The DFT results and vibrational assignments were summarized in Table S2.

Table S2. Vibrational assignments of DCM in the ground state

Experimental		Calculated[1]	Vibrational assignments[2,3]
in $CHCl_3$	*in* DMSO	*B3LYP/6-311G(d,p) PCM (DMSO)*	
-	1061	1044	$\delta_{CH3/CH}$ (py)
1170	1170	1144	$\nu_{9a} + \delta_{CH3}$ (ph)
1186	1190	1164	$\nu_{9a} + \delta_{CH3}$ (ph) + $\nu_{C\text{-}CH3}$ (py)
1212	1214	1192	ν_{9a} (ph) + δ_{CH} (py)
-	1275	1256	δ_{CH} (py)
1302	1308	1282	δ_{CH} (C_a=C_b)
1335	1339	1316	ν_3 (ph) + δ_{CH} (C_a=C_b)
1366	1363	1347	$\nu_{C\text{-}N} + \delta_{CH3/CH}$ (ph)
1387	1389	1370	δ_{CH3} (py)
1417	1417	1392	$\delta_{CH3} + \nu_{8b}$ (py)
-	1444	1417	δ_{CH3} (py)
1478	1476	1450	ν(C_c=C_d) + $\delta_{CH3/CH}$ (py)
1501	1497	1470	δ_{CH3} (ph)
1527	1527	1501	ν_{19b} (py) + δ_{CH3} (ph)
1552	1548	1512	$\nu_{19a} + \delta_{CH3}$ (ph)
1599	1600	1572	ν(C_a=C_b) + ν_{8a} (ph) (symmetric)
1620	1620	1605	ν(C_a=C_b) + ν_{8a} (ph) (asymmetric)
1652	1650	1623	ν_{8a} (py)
-	2190	2200	$\nu_{as,C\equiv N}$
2210	2207	2224	$\nu_{s,C\equiv N}$

[1] Calculated vibrational frequencies were rescaled with a factor of 0.967.[12]

[2] The ν and δ denote stretching and bending vibrations, respectively.

[3] The ph and py in parenthesis refer to the phenyl and pyran ring, respectively. The C_a=C_b represents the central olefinic double bond, and the C_c=C_d represents the double bond between the dicyanomethylene group and the pyran ring as shown in Figure S6.

Vibrational assignments of DCM in the excited state

The vibrational assignments of DCM in the excited states are considered challenging since the molecule faces substantial changes in the molecular structure, dipole moment, etc. in the excited state. In general, the molecular structure calculations in the excited state, for example, by the TDDFT methods are considered incomplete and need be improved substantially.[14-15] Nonetheless, we adopted the TDDFT simulations with the several hybrid functionals and basis sets for tentative vibrational assignments of the normal modes of DCM in the excited states. The molecular structures and Raman spectra of DCM in the LE and CT conformer states of the S_1 excited state were estimated by using the B3LYP/6-311G(d,p) and mPW1PBE/6-31G(d) simulations as described in the previous section. The molecular geometry of DCM in the LE ("*planar*" geometry) or the CT conformers ("*twisted*" along with the DMA or DMAP group) of the S_1 state obtained from these simulations do not show major differences between the exchange-correlation functionals or the basis sets used. As clearly shown in Figure S11, the differences in the simulated Raman spectra of DCM for the LE or CT conformers between the B3LYP/6-311G(d,p) and mPW1PBE/6-31G(d) simulation results are quite small except the $\nu_{C≡N}$ modes. The frequencies of the $\nu_{C≡N}$ modes of DCM obtained from the B3LYP/6-311G(d,p) simulations are strongly (~100 cm^{-1}) blue-shifted from those with the mPW1PBE/6-31G(d) simulations although the frequencies of other major vibrational modes are slightly red-shifted with the B3LYP functionals. In Figure S12, the simulated Raman spectra of DCM in the S_1 state were compared between the optimized geometries of the planar, twisted DMA and DMAP group obtained from the TDDFT simulations at the B3LYP/6-311G(d,p) and mPW1PBE/6-31G(d) levels. See Figure S8 and Table S1 for each optimized structures.

The $\nu_{C≡N}$ modes observed at 2170 cm^{-1} (LE) and 2175 and 2212 cm^{-1} (CT) in the transient Raman measurements showed not much changes in the simulated Raman spectra by TDDFT except the largely blue-shifted modes obtained from the B3LYP/6-311G(d,p) simulation

results with the twisted DMAP group. Since the changes in the relative intensities and vibrational assignments between the symmetric and asymmetric modes of $\nu_{C\equiv N}$ appeared not changed, the blue-shifts shown from the B3LYP/6-311G(d,p) simulation results with the twisted DMAP group would not be considered for the evidence of structure identification in the excited state. The red-shifts of $\nu_{C\equiv N}$ upon the ICT or charge separation in the excited states have been reported by time-resolved infrared absorption measurements.[16-18] It is interesting to note that another weak symmetric $\nu_{C\equiv N}$ band appears at 2212 cm^{-1} in the ICT state as shown in Figure 5. The appearance of the $\nu_{s,\,C\equiv N}$ mode which is invisible in the S_1/LE state or S_0 ground state may represent the changes in the molecular structure or symmetry (from "*planar*" to "*twisted*", e.g.) accompanying the ICT.

Based on the TDDFT simulations results, the excited state Raman bands of DCM in the S_1/LE state at 1436 and 1471 cm^{-1} are assigned as δ_{CH3} (ph) and δ_{CH3} (ph) + ν_{19b} (py), respectively. The major Raman bands in the S_1/LE state at 1563 (sh), 1580, and 1635 cm^{-1} are assigned as the symmetric and asymmetric $\nu(C_a{=}C_b)$ + ν_{8a} (ph), and ν_{8a} (py), respectively. The S_1/LE bands at 1156 and 1200 cm^{-1} are assigned as ν_{9a} + δ_{CH3} (ph) and ν_{9a} (ph), respectively, by considering the vibrational mode comparison between the bands at 1139 and 1186 cm^{-1} in the simulated spectrum for the S_1 state (planar) at B3LYP/6-311G(d,p) level and those at 1144 and 1192 cm^{-1} for the ground state. The ν_{9a} (ph) mode showed blue-shifts upon the ICT (1200 $\rightarrow$ 1212 cm^{-1}), which was not clearly explained by the DFT simulation results for both twisted structures of DMA or DMAP. Overall, the vibrational frequencies of DCM in the S_1 state (planar) seem to be slightly red-shifted from those obtained from the ground state geometry of DCM in the frequency range of 1500-2300 cm^{-1}. Another medium band at 1495 cm^{-1} in the LE spectrum of DCM in $CHCl_3$ (see Figure 3(a)) and at ~1500 cm^{-1} (sh) in the LE spectrum in DMSO (see Figure 2(a)) is assigned as the ν_{8b} (ph) mode. This band shows no major peak shifts

upon the rotation of the DMA or DMAP group in the excited state according to the TDDFT simulations.

The transient Raman bands of DCM in the S_1/CT state were also assigned based on the TDDFT simulation results. The vibrational bands appearing at 1057 and 1288 cm^{-1} in the CT spectrum was assigned as ν_{15} (ph) and ν_{14} (ph) mode, respectively, which is estimated to appear at 1090 (1095) and 1263 (1267) cm^{-1} for the twisted geometry of DMA group.[ii] The strong CT band at 1492 cm^{-1} which appeared blue-shifted during the ICT was assigned as ν_{19b} (py) + ν_{19a} (ph) mode, where the vibrational mode analysis and peak shift were better supported by the twisted geometry of the DMA group in the S_1 state. Another medium-strong CT band at 1428 cm^{-1} was assigned as ν_{19a} (ph) + δ_{CH} (py) mode. The skeletal vibrational modes at 1564 (sh), 1579, and 1635 cm^{-1} assigned as the symmetric and asymmetric $\nu(C_a{=}C_b)$ + ν_{8a} (ph), and ν_{8a} (py), respectively, for the LE structure, showed no major spectral changes with the DMA rotation. However, it is clear that the symmetrically and asymmetrically coupled vibrations of $\nu(C_a{=}C_b)$ and ν_{8a} (ph) modes break up into the decouple vibrational modes of $\nu_{C=C}$ + δ_{CH} $(C_a{=}C_b)$ and ν_{8a} (ph) at 1573 (1575) and 1616 (1621) cm^{-1} as predicted by the TDDFT simulations for the DMAP rotated conformers. The vibrational assignments for the transient Raman bands of DCM obtained in the S_1/LE and S_1/CT states were summarized in Table S3 in addition to the major vibrational frequencies of DCM obtained from the TDDFT simulations on the planar and "*twisted*" geometries of DMA and DMAP groups.

[ii] Frequencies outside the parentheses represent the simulated values at B3LYP/6-311G(d,p) level and those within the parentheses at mPW1PBE/6-31G(d) level.

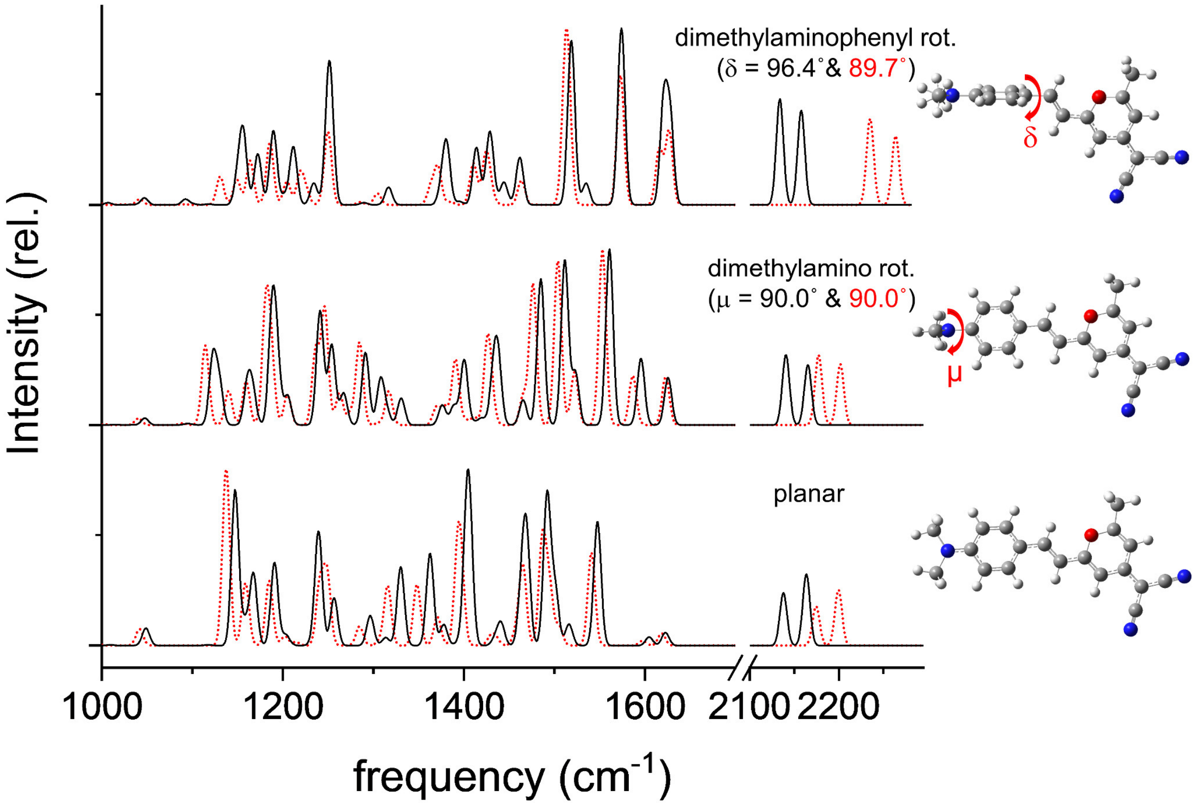

Figure S11. Simulated Raman spectra and optimized geometries of DCM in the S_1 excited state; the TDDFT simulations at the mPW1PBE/6-31G(d) level (solid lines)[10] and the B3LYP/6-311G(d,p) level (dotted lines) with PCM (DMSO).

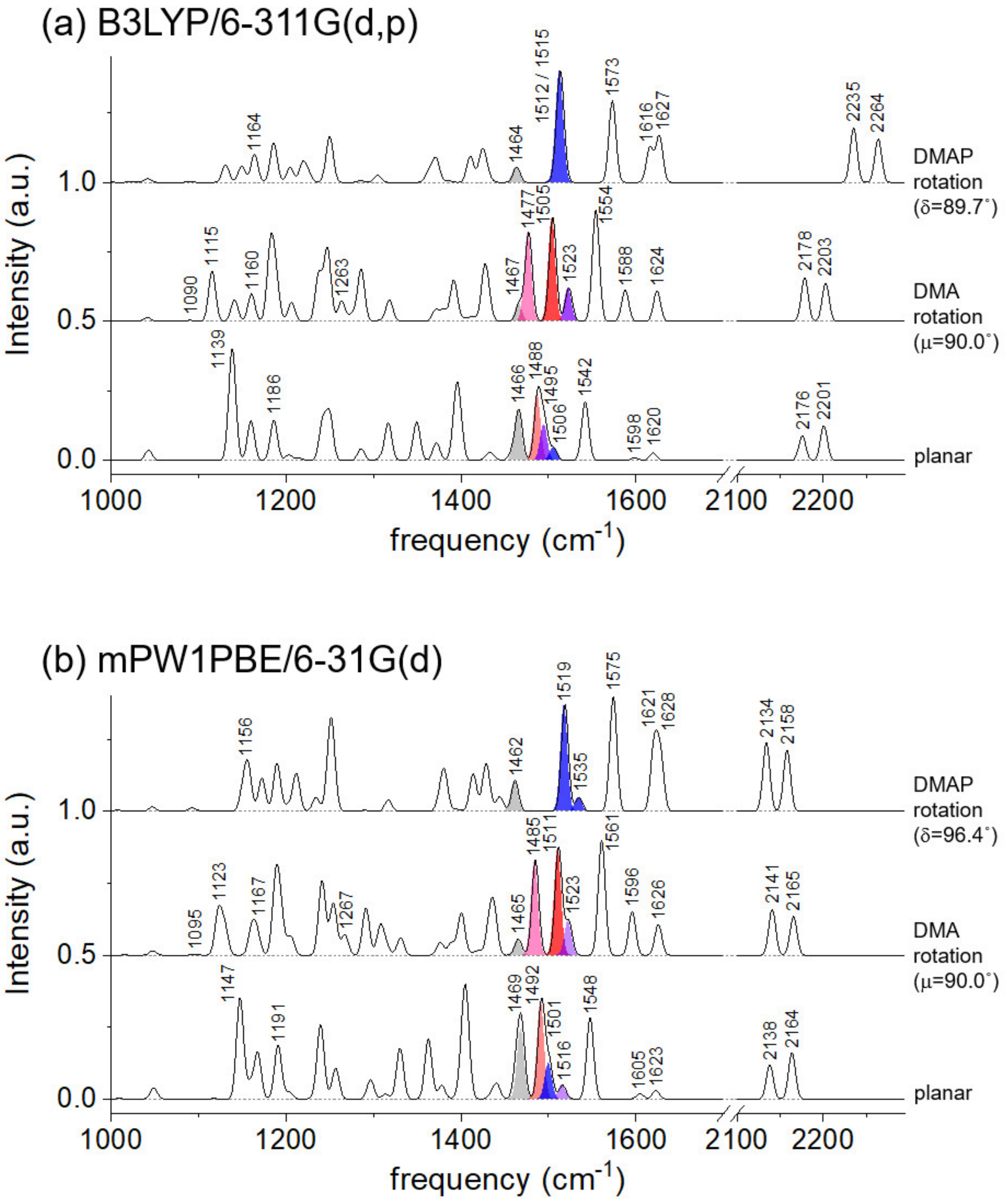

Figure S12. Calculated vibrational spectra for the optimized structures of DCM in the S_1 excited state from the DFT simulations at (a) B3LYP/6-311G(d,p) and (b) mPW1PBE/6-31G(d) levels. The spectral changes expected for ν_{19a}, ν_{19b}, or δ_{CH3} modes were displayed with color-codes.

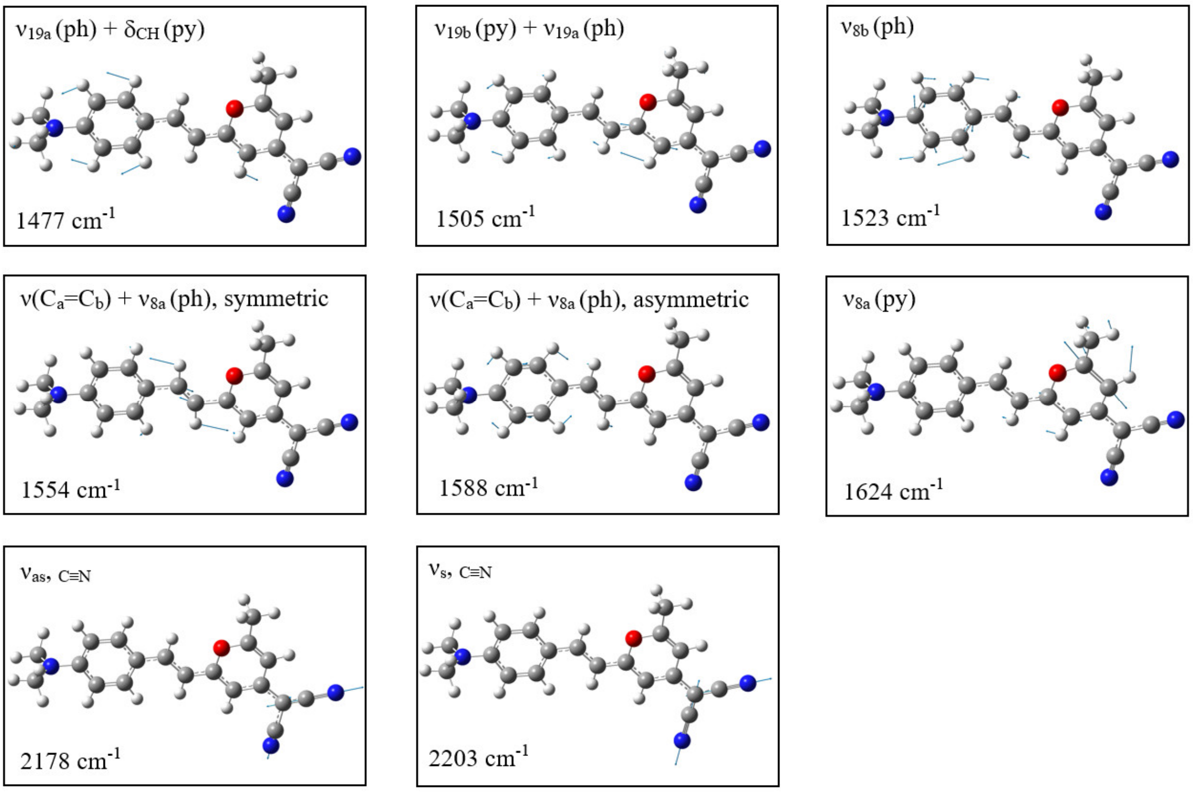

Figure S13. Vibrational normal modes of DCM with the twisted geometry of the dimethylamino (DMA) group (μ = 90.0°) obtained from the TD-B3LYP/6-311G(d,p) simulations with PCM (DMSO). The DFT results and vibrational assignments were summarized in Table S3.

Table S3. Comparison between the vibrational frequencies in the excited SRS of DCM and calculated vibrational frequencies from different structures of DCM

Experimental (DMSO)		B3LYP/6-311G(d,p)			mPW1PBE/6-31G(d)			Vibrational assignments[1,2]
LE (0.3 ps)	CT (30 ps)	planar	DMA rotated (μ=90.0°)	DMAP rotated (δ=89.7°)	planar	DMA rotated (μ=90.0°)	DMAP rotated (δ=96.4°)	
-	1057	-	1090	1089	-	1095	1092	ν_{15} (ph)
1156	1156	1139	1115	-	1147	1123	-	$\nu_{9a} + \delta_{CH3}$ (ph)
1200	1212	1186	1160	1164	1191	1167	1156	ν_{9a} (ph)
-	1288	-	1263	-	-	1267	-	ν_{14} (ph)
1436	-	1466	1467	1464	1469	1465	1462	δ_{CH3} (ph)
-	1428	-	1477	-	-	1485	-	ν_{19a} (ph) + δ_{CH} (py)
1471	-	1488	-	-	1492	-	-	δ_{CH3} (ph) + ν_{19b} (py)
-	1492	-	1505	-	-	1511	-	ν_{19b} (py) + ν_{19a} (ph)
1495[3]	-	1495	1523	-	1501	1523	-	ν_{8b} (ph)
-	-	1506	-	1512/1515	1516	-	1519	$\nu_{19a} + \delta_{CH3}$ (ph)
1563[sh]	1564[sh]	1542	1554	-	1548	1561	-	$\nu(C_{a=}C_b) + \nu_{8a}$ (ph), symmetric
-	-	-	-	1573	-	-	1575	$\nu_{C=C} + \delta_{CH}$ ($C_{a=}C_b$)
1580	1579	1598	1588	-	1605	1596	-	$\nu(C_{a=}C_b) + \nu_{8a}$ (ph), asymmetric
-	-	-	-	1616	-	-	1621	ν_{8a} (ph)
1635	1635	1620	1624	1627	1623	1626	1628	ν_{8a} (py)
2170	2175	2176	2178	2235	2138	2141	2134	$\nu_{as,C\equiv N}$
-	2212	2201	2203	2264	2164	2165	2158	$\nu_{s,C\equiv N}$

[1] Vibrational assignments are based on calculated vibrational frequencies in the excited state at the B3LYP/6-311G (d,p) and mPW1PBE/6-31G(d) levels, and frequencies were rescaled with a factor of 0.967.[12]

[2] ν and δ denotes stretching and bending, respectively. The ph and py in parenthesis refer to the benzene and pyran ring, respectively, and C_a=C_b represents the ethylenic double bond as shown in Figure S6.

[3] observed from $CHCl_3$ solution

[sh] shoulder band

4. FSRS of 4-dimethylamino-4′-nitrostilbene

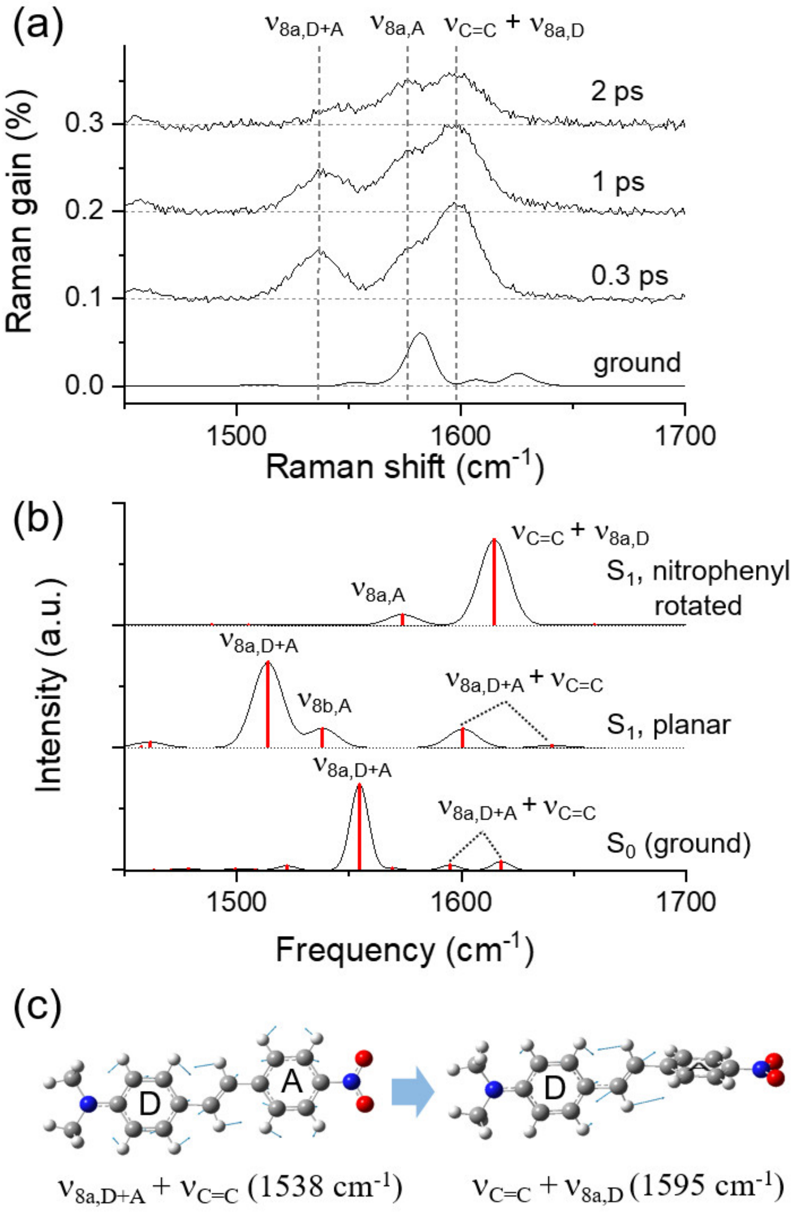

Figure S14. (a) FSRS of 4-dimethylamino-4′-nitrostilbene in $CHCl_3$ with 403 nm excitation, (b) simulated Raman spectra for the optimized structures in the ground and excited states by the (TD)DFT method at B3LYP/6-311G(d,p) level, and (c) the major vibrational modes of $\nu_{C=C}$ and ν_{8a} of phenyls for the planar and twisted nitrophenyl conformers in the excited state (adapted from ref. 19-20).

5. References

1. Lee, S.; Lee, J.; Pang, Y. Excited state intramolecular proton transfer of 1,2-dihydroxyanthraquinone by femtosecond transient absorption spectroscopy. *Curr. Appl. Phys.* **2015,** *15*, 1492-1499.
2. Lee, J.; Pang, Y. Metal-Enhanced Fluorescence: Ultrafast Energy Transfer from Dyes in a Polymer Film to Metal Nanoparticles. *J. Nanosci. Nanotech.* **2016,** *16* (2), 1629-1632.
3. Lee, J.; Lee, S.; Jen, M.; Pang, Y. Metal-enhanced fluorescence: Wavelength-dependent ultrafast energy transfer. *J. Phys. Chem. C* **2015,** *119*, 23285-23291.
4. Lee, I.; Lee, S.; Pang, Y. Excited-State Dynamics of Carotenoids Studied by Femtosecond Transient Absorption Spectroscopy. *Bull. Korean Chem. Soc.* **2014,** *35* (3), 851-857.
5. Jen, M.; Lee, S.; Jeon, K.; Hussain, S.; Pang, Y. Ultrafast Intramolecular Proton Transfer of Alizarin Investigated by Femtosecond Stimulated Raman Spectroscopy. *J. Phys. Chem. B* **2017,** *121*, 4129-4136.
6. van Stokkum, I. H. M.; Larsen, D. S.; Van Grondelle, R. Global and target analysis of time-resolved spectra. *Biochim. Biophys. Acta* **2004,** *1657*, 82-104.
7. Snellenburg, J. J.; Laptenok, S. P.; Seger, R.; Mullen, K. M.; Stokkun, I. H. M. Glotaran : A Java -Based Graphical User Interface for the R Package TIMP. *J. Stat. Softw.* **2012,** *49*, 1-22.
8. Gustavsson, T.; Baldacchino, G.; Mialocq, J. C.; Pommeret, S. A femtosecond fluorescence up-conversion study of the dynamic Stokes shift of the DCM dye molecule in polar and non-polar solvents. *Chem. Phys. Lett.* **1995,** *236* (6), 587-594.
9. Frisch, M. J.; Trucks, G. W.; Schlegel, H. B.; Scuseria, G. E.; Robb, M. A.; Cheeseman, J. R.; Scalmani, G.; Barone, V.; Mennucci, B.; Petersson, G. A.; Nakatsuji, H.; Caricato, M.; Li, X.; Hratchian, H. P.; Izmaylov, A. F.; Bloino, J.; Zheng, G.; Sonnenberg, J. L.; Hada, M.; Ehara, M.; Toyota, K.; Fukuda, R.; Hasegawa, J.; Ishida, M.; Nakajima, T.; Honda, Y.; Kitao, O.; Nakai, H.; Vreven, T.; Montgomery, J. A.; Peralta, J. E.; Ogliaro, F.; Bearpark, M.; Heyd, J. J.; Brothers, E.; Kudin, K. N.; Staroverov, V. N.; Kobayashi, R.; Normand, J.; Raghavachari, K.; Rendell, A.; Burant, J. C.; Iyengar, S. S.; Tomasi, J.; Cossi, M.; Rega, N.; Millam, J. M.; Klene, M.; Knox, J. E.; Cross, J. B.; Bakken, V.; Adamo, C.; Jaramillo, J.; Gomperts, R.; Stratmann, R. E.; Yazyev, O.; Austin, A. J.; Cammi, R.; Pomelli, C.; Ochterski, J. W.; Martin, R. L.; Morokuma, K.; Zakrzewski, V. G.; Voth, G. A.; Salvador, P.; Dannenberg, J. J.; Dapprich, S.; Daniels, A. D.; Farkas; Foresman, J. B.; Ortiz, J. V.; Cioslowski, J.; Fox, D. J. Gaussian 09, Revision B.01. Wallingford CT, 2009.

10. Xu, X.; Zhang, R.; Cao, Z.; Zhang, Q. Intramolecular charge transfer and photoisomerization if the DCM styrene dye: a theoretical study. *J. Theor. Comput. Chem.* **2008,** *7*, 719-36.
11. Nabavi, S. H.; Khodabandeh, M. H.; Golbabaee, M.; Moshaii, A.; Davari, M. D. Excited states study reveals the twisted geometry induced large stokes shift in DCM fluorescent dye. *J. Photochem. Photobiol. A* **2018,** *354*, 127-138.
12. Irikura, K. K.; Johnson, R. D.; Kacker, R. N. Uncertainties in Scaling Factors for ab Initio Vibrational Frequencies. *J. Phys. Chem. A* **2005,** *109* (37), 8430-8437.
13. Merrick, J. P.; Moran, D.; Radom, L. An Evaluation of Harmonic Vibrational Frequency Scale Factors. *J. Phys. Chem. A* **2007,** *111* (45), 11683-11700.
14. Eriksen, J. J.; Sauer, S. P. A.; Mikkelsen, K. V.; Christiansen, O.; Jensen, H. J. A.; Kongsted, J. Failures of TDDFT in describing the lowest intramolecular charge-transfer excitation in para-nitroaniline. *Mol. Phys.* **2013,** *111* (9-11), 1235-1248.
15. Elliott, P.; Furche, F.; Burke, K. Excited states from time-dependent density functional theory. In *Rev. Comput. Chem.*, Lipkowitz, K. B.; Cundari, T. R., Eds. John Wiley & Sons, Inc.: 2009; pp 91-165.
16. Dereka, B.; Rosspeintner, A.; Krzeszewski, M.; Gryko, D. T.; Vauthey, E. Symmetry-Breaking Charge Transfer and Hydrogen Bonding: Toward Asymmetrical Photochemistry. *Angew. Chem. Int. Ed.* **2016,** *55* (50), 15624-15628.
17. Dereka, B.; Koch, M.; Vauthey, E. Looking at Photoinduced Charge Transfer Processes in the IR: Answers to Several Long-Standing Questions. *Acc. Chem. Res.* **2017,** *50* (2), 426-434.
18. Mani, T.; Grills, D. C. Nitrile Vibration Reports Induced Electric Field and Delocalization of Electron in the Charge-Transfer State of Aryl Nitriles. *J. Phys. Chem. A* **2018,** *122* (37), 7293-7300.
19. Lee, S.; Song, J.; Jen, M.; Pang, Y. Twisted Intramolecular Charge Transfer of Nitroaromatic Molecules Studied by Femtosecond Stimulated Raman Spectroscopy. *manuscript in preparation*.
20. Lee, S.; Jen, M.; Lee, G.; Pang, Y. In *Structural Changes of Nitroaromatic Molecules During the Intramolecular Charge Transfer*, Frontiers in Optics / Laser Science, Washington, DC, 2020/09/17; Optical Society of America: Washington, DC, 2020; p FTh2D.5.
